# Elevated CO_2_ Increases the Canopy Temperature of Mature *Quercus robur* (Pedunculate Oak)

**DOI:** 10.1111/gcb.70565

**Published:** 2025-11-05

**Authors:** William Hagan Brown, Emanuel Gloor, Ralph Fyfe, A. Rob MacKenzie, Nicholas J. Harper, Peter Ganderton, Kris Hart, Giulio Curioni, Susan Quick, Scott J. Davidson, Emily Yetton, Jen L. Diehl, Sophie Fauset

**Affiliations:** ^1^ School of Geography, Earth and Environmental Sciences University of Plymouth Plymouth UK; ^2^ CSIR‐Forestry Research Institute of Ghana Kumasi Ghana; ^3^ School of Geography, Faculty of Environment University of Leeds Leeds UK; ^4^ Birmingham Institute of Forest Research, and School of Geography, Earth and Environmental Sciences University of Birmingham Birmingham UK; ^5^ Forest Research, Climate Change Group Northern Research Station Roslin Midlothian UK; ^6^ Center for Ecosystem Science and Society (ECOSS) Northern Arizona University Flagstaff Arizona USA; ^7^ School of Informatics, Computing, and Cyber Systems (SICCS) Northern Arizona University Flagstaff Arizona USA

**Keywords:** carbon dioxide, climate change, free‐air CO_2_ enrichment (FACE), leaf traits, microclimate, stomata, stomatal conductance, temperate forests

## Abstract

The canopy thermal response of natural forests to elevated CO_2_ (eCO_2_) is an understudied biophysical feedback in the global climate system. We investigated the effects of eCO_2_ (150 μmol mol^−1^ above ambient) on canopy temperature (*T*_can_) dynamics of mature (> 175 years) *Quercus robur* (oak) at the Birmingham Institute for Forest Research Free Air CO_2_ Enrichment (BIFoR‐FACE) facility in Staffordshire, England, during the growing seasons of 2021, 2022 and 2023. We employed long‐term, high‐frequency thermal infrared (TIR) imaging to measure *T*_can_. Our results show that daily maximum oak *T*_can_ under eCO_2_ was, on average, approximately 1.3°C higher than under ambient (aCO_2_) conditions (21.5°C ± 4.4°C for aCO_2_ vs. 22.8°C ± 5.2°C for eCO_2_ oaks). Moreover, daily maximum *T*_can_–air temperature (*T*_air_) differences were significantly higher under eCO_2_, resulting from more frequent extreme temperature excursions. These differences appear primarily to be driven by reduced stomatal conductance under eCO_2_, which limits transpirational cooling and alters the surface energy balance. This effect was evident in the different relationship between *T*_can_–*T*_air_ and vapour pressure deficit (VPD) for eCO_2_ compared to aCO_2_, showing a reduction in transpirational cooling under high VPD. Also, CO_2_‐induced leaf structural and anatomical modifications, such as increased leaf mass per area, may have enhanced solar radiation absorption, thereby enabling greater canopy warming under high radiation conditions. Thus, eCO_2_ could likely cause a reduction in leaf transpiration in oaks, reducing its contribution to processes such as humidification of the lower atmosphere and precipitation in local and regional climates. Our findings highlight how high CO_2_ conditions may intensify thermal stress in temperate forests, influencing water and carbon cycles and potentially impacting forest resilience. Furthermore, *T*_can_ will be essential for refining global Earth system models, which often use *T*_air_ as a proxy for *T*_can_, despite the latter's direct influence on carbon and hydrological cycles.

## Graphical abstract

We investigated the impact of high atmospheric CO_2_, similar to that predicted for 2050, on tree canopy temperature dynamics of mature pedunculate oak using long‐term, high‐frequency thermal infrared imaging. Canopies under elevated CO_2_ consistently exhibited higher leaf temperatures than those under ambient conditions. This warming may be linked to reduced transpirational cooling due to lower stomatal conductance and increased leaf thickness. Our findings demonstrate that elevated CO_2_ may exacerbate thermal stress in temperate forests, with potential consequences for the water cycle, carbon uptake, and forest resilience in a warming climate.

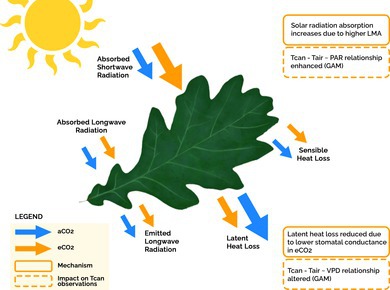

## 1 Introduction

Forest canopies are vital to the Earth's system because they are a primary site where carbon fixation occurs. This process represents one of the largest carbon fluxes in the Earth system, contributing significantly to carbon storage (Bonan 2008; Friedlingstein et al. 2022; Pan et al. 2024). In addition to being carbon sinks when net ecosystem exchange is positive, forest canopies also support biodiversity conservation and play an essential role in climate change mitigation (Lowman and Nadkarni 1995; Mitchell et al. 2002). Forest canopies play a key role at the biosphere‐atmosphere interface, significantly impacting both the hydrological and carbon cycles (Mitchell et al. 2002; Ozanne et al. 2003). Indeed, the sun‐lit leaves of the upper canopy predominantly process a forest's carbon uptake, impacting many ecosystem processes vital for forest health (Campbell and Norman 1998; Doughty and Goulden 2008; Lowman and Nadkarni 1995). While forest canopies act as a direct buffer for terrestrial ecosystems against the extreme effects of climate change, they are increasingly at risk of warming (Doughty et al. 2023; O'Sullivan et al. 2017). Increased canopy temperatures may jeopardize overall forest health and function, rendering species that benefit from these niches vulnerable to extreme climatic impacts (Kim et al. 2022). Thus, understanding canopy‐top temperature (*T*_can_; defined here as the aggregated temperature of leaf assemblages at the scale of branches to crowns as per Still et al. 2021) dynamics is crucial, given its significant role in shaping the overall forest ecosystem by regulating transpiration and the exchange of energy (Bannister et al. 2022; Monteith and Unsworth 2013).

**FIGURE 1:**
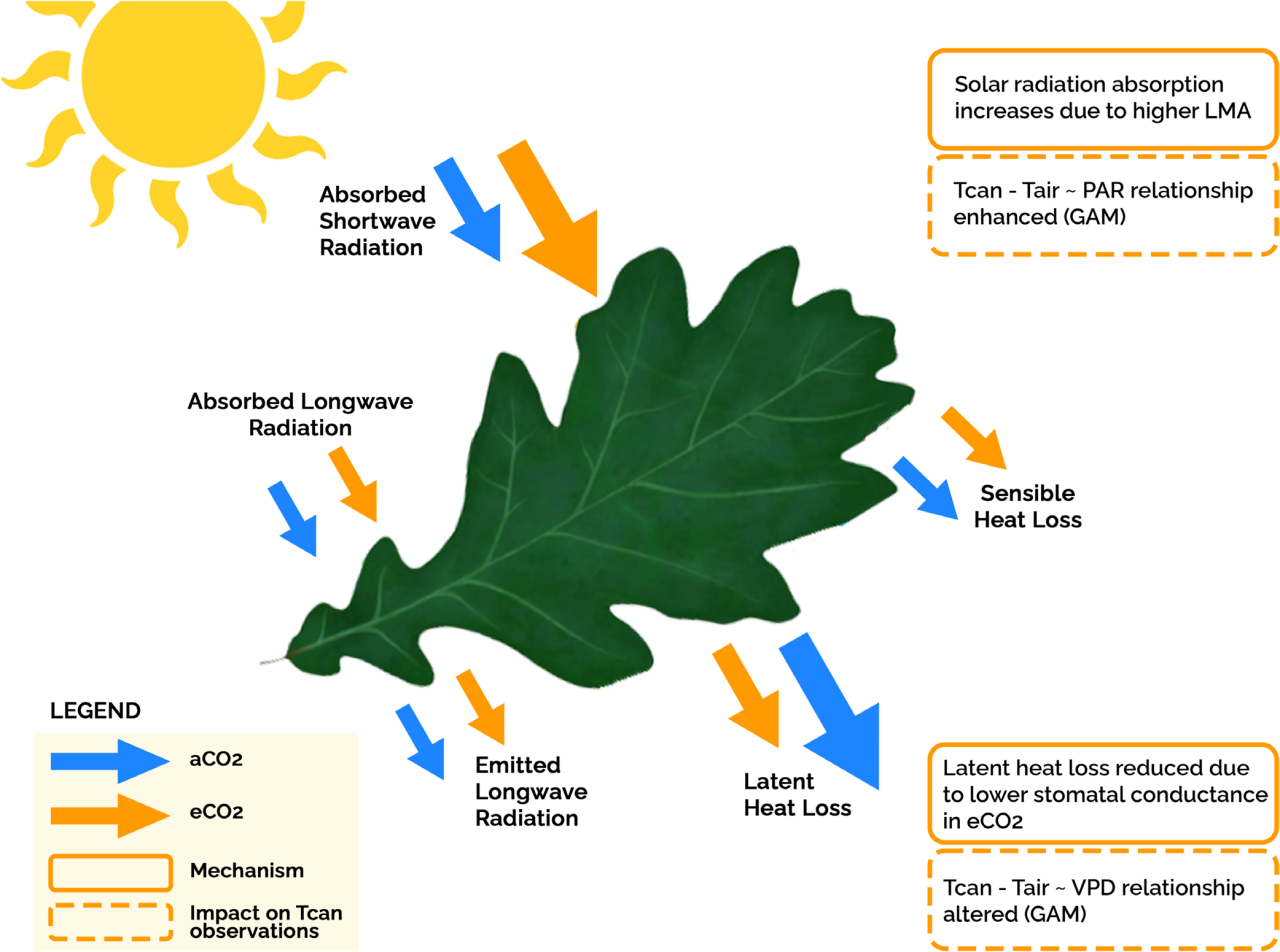
Leaf energy balance schematic illustrating the hypothesised effects of elevated CO_2_ on leaf temperature at BIFoR FACE. The size of each arrow represents the relative magnitude of the associated mechanism under the respective treatment or condition. The same arrow sizes are used for other processes (absorbed/emitted longwave radiation and sensible heat loss) that influence leaf temperature, which were not expected to be influenced by elevated CO_2_ in this study.

Leaf temperature (*T*_leaf_) directly modulates photosynthesis and respiration because it alters cell‐membrane fluidity, enzyme kinetics, and the solubility and diffusivity of CO_2_ and O_2_ (Jones 2014). Thermal imaging and energy balance studies of leaves spanning tropical to temperate forests reveal that *T*_can_ is usually several degrees warmer than the surrounding air (Still et al. 2022). Indeed, average daytime leaf‐to‐air offsets of 2°C–5°C are common, with excursions above 10°C occurring under conditions of high radiation and vapour‐pressure deficit (Fauset et al. 2018; Still et al. 2021). This canopy‐to‐air temperature divergence arises because the absorbed short and long‐wave radiation cannot be fully dissipated through cooling processes (sensible heat or as latent heat loss) sufficient to effectively cool the leaves to air temperature. These findings suggest that, as global warming persists, there is potential for both air and leaf temperatures to exceed the optimal photosynthetic temperatures of species, thereby reducing carbon assimilation, growth rates, and tree survival (Crous et al. 2023; Sage et al. 2008; Still et al. 2022). Quantifying how high CO_2_ modifies *T*_can_ is a necessary first step in assessing the leaf‐energy balance in a changing climate and, hence, for predicting how forests will respond (Figure 1).

Understanding the response of plants to elevated CO_2_ (eCO_2_) under natural environmental conditions has been achieved through Free‐Air‐Carbon‐Enrichment (FACE) studies (Ainsworth and Long 2005; Leuzinger and Körner 2007; Norby et al. 2010). FACE studies across significant temporal and spatial scales have shown that higher CO_2_ levels often increase photosynthesis in C3 plants, enhancing growth and yield (Ainsworth and Long 2021; Norby et al. 2010). In addition, FACE experiments have reported reduced stomatal conductance in C3 plants under eCO_2_ (Bernacchi et al. 2007; Herrick et al. 2004; Liang et al. 2023). Reduced stomatal conductance under eCO_2_ often has a more pronounced impact on transpiration than on photosynthesis (Bernacchi et al. 2007), increasing water‐use efficiency but also leaf temperature, especially during high‐temperature conditions (Birami et al. 2020; Hui et al. 2001; Warren, Pötzelsberger, et al. 2011; Zhu et al. 2017).

While FACE studies involving mature trees are limited, evidence from crops, saplings, and the tree‐level studies also consistently point to reduced canopy water loss under eCO_2_ (Ainsworth and Long 2005; Bernacchi et al. 2007; Leakey et al. 2009). A meta‐analysis of 13 European tree studies reported a 21% reduction in stomatal conductance under eCO_2_, with younger, deciduous, and water‐stressed trees exhibiting stronger reductions than mature or less stressed species (Medlyn et al. 2001). Ongoing FACE research on mature *Quercus robur* L. ( *Q. robur* ) forests at the Birmingham Institute of Forest Research (BIFoR) reported a 7% reduction in stomatal conductance, a 33% ± 8% increase in light‐saturated photosynthesis in top canopy leaves with potential implications for improved water use efficiency and reduced transpiration (Gardner et al. 2021; Gardner et al. 2023). Tree‐scale sap flow studies also corroborate these leaf‐level trends at BIFoR‐FACE. For instance, Quick et al. (2025) reported year‐dependent but consistent 4%–16% declines in daily water use in oaks under eCO_2_ conditions. Comparable reductions of 7%–16% were observed in the ORNL‐FACE sweetgum plantation, and Web‐FACE reported a 14% decrease in sap flow of temperate beech and hornbeam (Leuzinger and Körner 2007; Warren, Pötzelsberger, et al. 2011).

Along with short‐term stomatal conductance responses, eCO_2_‐induced alterations of stomatal anatomy (size and density) and anatomical maximum conductance have been reported (Hetherington and Woodward 2003; Lawson and Blatt 2014; Lawson and Vialet‐Chabrand 2019). However, such long‐term responses in FACE and natural eCO_2_ systems are not always the same. In some cases, such as a natural CO_2_ spring study (Bettarini et al. 1998) and a 5‐year poplar FACE trial (Tricker et al. 2005), stomatal conductance remained lower under eCO_2_ without lasting changes in stomatal density. Meta‐analysis confirms this pattern: eCO_2_ reduced stomatal conductance by about 22% on average (Ainsworth and Rogers 2007), yet stomatal density changes were small and inconsistent across species, with no general trend in size. These findings suggest that long‐term stomatal conductance is maintained mainly through instantaneous stomatal conductance dynamics rather than persistent stomatal anatomical changes (Saban et al. 2019). Thus, at BIFoR FACE, lower stomatal conductance under eCO_2_ is expected to reduce latent heat loss, leading to higher leaf temperatures and an increased canopy‐to‐air temperature difference (*T*_can_–*T*_air_). This thermal response may occur independently of changes in stomatal morphology. Also, because stomatal conductance is highly sensitive to VPD, altered stomatal dynamics would modify the *T*_can_–*T*_air_ VPD relationship with implications for the surface energy balance and regional water cycles (Kovenock and Swann 2018; Leuzinger and Körner 2007; Li 2024; Figure 1).

FACE studies have also demonstrated that eCO_2_ leads to altered leaf morphological traits that govern leaf energy balance. Variations in leaf traits such as width and thickness modulate solar energy absorption, heat dissipation, and leaf temperature (Fauset et al. 2018; Vinod et al. 2023). Under eCO_2_, trees commonly show higher leaf mass per area (LMA) in upper‐canopy leaves, indicating thicker, darker foliage (Norby et al. 2021). Indeed, at BIFoR FACE, 2019–2020 measurements reported a 16% increase in LMA under eCO_2_ (Foyer et al. 2025; Gardner et al. 2022). Increased leaf mass per area (LMA) in upper canopy leaves can increase leaf thermal mass and absorption (Kovenock and Swann 2018; Ollinger 2010; Zhou et al. 2023). Such leaf structural changes could thus result in increased heat capacity, which in turn influences the energy balance and thermoregulation of the tree canopy leaves (Bonan 2008; Leuzinger and Körner 2007; Figure 1).

The most direct approach to measuring leaf temperature uses point measurements such as leaf thermistors and thermocouples, which can provide high temporal resolution data for a few leaves (Fauset et al. 2018; Kim et al. 2018; Farella et al. 2022). While point measurements provide valuable species‐specific data on leaf energy balance (Doughty and Goulden 2008), their spatial and temporal limitations constrain forest stand and ecosystem application (Farella et al. 2022). Thermal infrared (TIR) cameras offer a promising solution by capturing surface temperatures across entire canopies, enabling comprehensive and continuous monitoring of individual trees and forest ecosystems (Aubrecht et al. 2016; Farella et al. 2022; Mayanja et al. 2024).

Recent applications of TIR imaging in natural forest ecosystems have enabled the detection of tree species' stomatal response to atmospheric dryness and transpiration rates, shedding light on the physiological processes affecting plant productivity (Kim et al. 2016; Leuzinger and Körner 2007; Lin et al. 2017; Still et al. 2021; Yi et al. 2020). Leveraging the power of TIR cameras in long‐term FACE applications will thus improve our understanding of forest thermal responses and the ecological impacts of eCO_2_, whilst capturing transient extreme climatic events during such long‐term experiments.

This study uses TIR imaging to obtain high–temporal resolution measurements of *T*_can_ in mature oaks under ambient (aCO_2_) and eCO_2_. We aim to determine whether there has been an increase in mature oak canopy temperature due to eCO_2_ impacting the leaf energy balance (Figure 1, energy balance schematic). We further aim to gain insight into changes in leaf energy balance by examining shifts in the relationship between canopy‐to‐air temperature differences and microclimate variables such as photosynthetic active radiation (PAR), vapour pressure deficit (VPD), soil volumetric water content (VWC), wind speed (WS), and whether these relationships differ between treatments.

*Quercus robur* is a member of the largest genus in the Fagaceae family, a genus that is very geographically widespread and among the largest genera of all tree families (Carrero et al. 2020). It is one of the most common broadleaf tree species with a widespread distribution across Europe, where it is both economically and ecologically important. Thus, conducting climate change impact experiments on *Q. robur* , among other species, provides an opportunity for understanding, modeling, and planning adaptation and mitigation strategies for temperate forest communities.

Our specific objectives with this study are to:

1. Determine the impact of eCO_2_ on *T*_can_ of mature *Q. robur* .
2. Quantify changes in leaf thermal traits under eCO_2_.
3. Examine changes in canopy‐to‐air temperature difference (*T*_can_–*T*_air_) in relation to micro‐climatic factors under different CO_2_ treatments as a consequence of altered leaf energy balance.

## 2 Materials and Methods

### 2.1 Study Site and FACE Experiment Description

Measurements of canopy temperatures were undertaken at the Birmingham Institute of Forest Research (BIFoR) Free Air CO_2_ Enrichment (FACE) facility located near Staffordshire in central England (52.801° N, 2.301° W), United Kingdom (Figure S1). The 19.1 ha site is situated within a temperate, deciduous forest dominated in the upper canopy by mature (> 175 years) pedunculate oak ( *Q. robur* ) (Gardner et al. 2021). The understory consists of dense vegetation, mostly of hazel coppice ( *Corylus avellana* L.), sycamore ( *Acer pseudoplatanus* L.), and hawthorn ( *Crataegus monogyna* Jacq.). The climate is characterised by cool, wet winters and warm summers, with the growing season (April to October) remaining frost‐free (Gardner et al. 2021).

The overarching BIFoR FACE setup (Figure S1) employs an *n* = 3 design, comprising six infrastructure arrays (plots) composed of three eCO_2_ treatment plots and three aCO_2_ control plots, along with three non‐infrastructure (ghost) plots, as described in Hart et al. (2020). Each control and treatment infrastructure plot consists of sixteen 25‐m tall steel structures, encompassing a woodland plot measuring approximately 30 m in diameter and extending above the local tree canopy. The air of the aCO_2_ plots has a CO_2_ mole fraction of approximately 405 μmol mol^−1^ at the start of the experiment in April 2017. Treatment eCO_2_ plots were supplied with air with aCO_2_ of +150 μmol mol^−1^ CO_2_ during the daylight hours when solar elevation was above −6.5° (Hart et al. 2020).

### 2.2 TIR Camera System Set‐Up and Operation

A Fluke RSE300 TIR camera (Fluke Corporation, Everett, WA, USA), with 360 × 240‐pixel resolution, was used to measure canopy temperatures. The camera, equipped with an uncooled microbolometer detector, measures longwave spectra in the 8–14 μm range. It operates within a temperature range of −10°C to +50°C and relative humidity of 10%–95% (non‐condensing). The camera unit was housed in an aluminium shield to protect against direct radiation and rain. The TIR camera was mounted on a 40 m flux tower (Figure S1), facing north, and inclined at 20°–25° below the horizon to minimise viewing geometry effects on TIR measurements. The TIR camera was connected via Ethernet to a desktop computer at the base of the flux tower, and LABVIEW (National Instruments, Austin, TX, USA) software was used for camera control.

This study focused on mature oaks in an eCO_2_ plot (‘array [A] 6’) and an adjacent aCO_2_ non‐infrastructure “ghost” plot (‘array [A] 7’); Figures S1 and S2. These plots were selected for treatment and control comparisons due to their proximity and shared TIR camera field of view (FoV). CO_2_ concentration is monitored in all the plots, and data show there is minimal diffusion of CO_2_ into control (aCO_2_ and Ghost) patches because the mixing of CO_2_ away from the treatment patches is strongly directed towards the vertical (Bannister et al. 2023; Harper 2023). The cameras were deployed in July 2021, capturing canopy images at a 10‐min resolution, resulting in a maximum of 144 images daily under ideal conditions. Despite disruptions in continuous logging due to malfunctions in the camera control software, the study achieved a high temporal resolution by collecting data for approximately 454 days: 116 days in 2021, 184 days in 2022, and 154 days in 2023. The full dataset is publicly available (Fauset et al. 2025). 

### 2.3 Canopy Temperature Data Retrieval, Correction, and Calibration

The retrieval, correction, and calibration of canopy temperature data followed the protocols outlined by Aubrecht et al. (2016). Polygons representing regions of interest (ROIs; Figures S1 and S2) in sunlit canopy areas of eCO_2_ and aCO_2_ plots were digitised using raw TIR images recorded on clear, sunny days at the time of full canopy closure. During ROI selection, care was taken to avoid selecting tree canopy gaps, non‐target species, and woody tissues, and to exclude lattice towers surrounding the treatment plots, thereby reducing artefacts and canopy motion effects on static ROIs. The distance between the TIR camera and the canopy ROIs ranged between 63 and 68 m, with each TIR image pixel encompassing three to four leaves at this distance. Four polygons were created, each for eCO_2_ and aCO_2_ plots, and each ROI measured approximately 20 cm × 20 cm at ~70 m from the camera. For each 10‐min image, the maximum pixel value within each ROI was extracted, and these maximum values were then averaged across all ROIs within each treatment to derive a single *T*_can_ value per treatment per image for further analysis.

Accurate TIR temperature measurement requires corrections for atmospheric attenuation, relative humidity, and reflected background radiation. Corrections applied at the BIFoR FACE site followed a three‐step procedure described by Aubrecht et al. (2016). First, the camera's default leaf emissivity and background temperature settings were recalibrated using local meteorological data, ensuring that the total measured radiation aligns with real field conditions. Here, “background” refers to the camera's assumed temperature of the surrounding environment. Second, pixel‐level atmospheric transmission corrections were applied using relative humidity, air temperature, and the distance between the camera and the canopies to account for radiation attenuation. Finally, these atmospheric transmission values were used in the equation from Aubrecht et al. (2016) to compute canopy surface temperatures for each pixel in the TIR images.

(1) $$\Phi_{\text{leaf}}=\frac{1}{{\tau \varepsilon }_{\text{leaf}}}\left(\Phi_{\mathrm{tot}}-\tau \left(1-\varepsilon_{\text{leaf}}\right)\varepsilon_{\mathrm{sky}}\Phi_{\mathrm{sky}}-\left(1-\tau \right)\Phi_{\mathrm{air}}\right)$$

*Φ*_leaf_ is the thermal energy radiated by the target tree canopy, while *Φ*_sky_ is the energy from thermal energy emitted by the sky and reflected off the canopy, and *Φ*_air_ is the energy added by the air between the canopy and the TIR camera. *ε*_leaf_ and *ε*_sky_ are the emissivities of the tree canopy and the sky, respectively, and *τ* is the transmission of air between the tree canopy and the thermal camera and accounts for the attenuation of thermal signals by water vapour in air.

Because thermal sensors can experience systematic drift, studies have often used a high‐emissivity reference plate to control and, if needed, compensate for such effects (Kim et al. 2018; Muller et al. 2021). Following this approach, the corrected TIR data were calibrated against a custom‐built 60 × 60 × 0.1 cm aluminium reference plate, which was roughened and painted matte black (emissivity ~0.99) (Aubrecht et al. 2016; Kim et al. 2016). In August and September of 2022, two pre‐calibrated copper‐constantan J‐type thermocouples were attached to the back of the plate, and the plate was mounted in the field of view of both the visible and TIR cameras.

Reference plate temperature data were extracted using the same procedures employed for canopy data extraction. A single 4 × 4 pixel ROI was digitised from TIR images of the reference plate, and mean temperatures were calculated for each 10‐min timestamp. A quadratic regression (Figure S4A,B) of thermocouple (reference plate) measurements against TIR‐derived plate data provided the calibration equation for corrected TIR canopy temperatures. The workflow for correcting the raw TIR camera data (Figure S3) is provided in the Supporting Information.

To further test for any artefacts of our use of a ghost array (with no infrastructure) to represent the aCO_2_ treatment rather than an ambient infrastructure array, thermistors (Ecomatik LAT‐B3, Munich, Germany) were attached to two leaves in the infrastructure aCO_2_ control array A5 and two leaves in the eCO_2_ array A6 during September 2023. We assessed whether the eCO_2_ treatment effects on *T*_can_–*T*_air_ and the relationship between *T*_can_ and *T*_air_ using our thermal camera data were similar to those shown by the data of *T*_leaf_ and *T*_air_ as measured with the thermistors. This comparison shows that the results we observe using the TIR camera are also found using leaf‐level measurements, with a tendency to see a larger effect size using the thermistor data, so our findings may be a conservative estimate of the actual CO_2_ effect (Figures S6 and S7).

### 2.4 Leaf Thermal Trait Measurements

Data on leaf structural traits, leaf area (LA) and leaf mass per area (LMA) were obtained from routine monthly leaf samples collected from defined canopy positions: top/sunlit (top 2 m, generally 20–25 m) and bottom (8–10 m) for a typical 25 m *Quercus robur* at BIFoR FACE (Gardner et al. 2022) during June to August of 2021, 2022, and 2023. These measurements were based on samples of 15 fully expanded leaves from 12 trees (six trees per aCO_2_ and eCO_2_ treatment) collected by arborists. Leaves were scanned using a LI‐3100C leaf area meter (LICOR Biosciences, USA) to calculate the LA. The leaves were then oven‐dried at 70°C for at least 72 h, and the dry weight was determined. The LMA was calculated as the ratio of the dry weight of the leaves to the LA.

Leaf stomatal density and morphology for eCO_2_ and aCO_2_ leaves were examined using leaf epidermal impression methods described in Franks et al. (2014). Stomatal density and size (product of stomatal length and width) are important determinants of maximum stomatal conductance and can be used to infer potential leaf cooling capacity and the dynamics of stomatal opening under changing environmental conditions (Drake et al. 2013; Franks and Beerling 2009; Lawson and Blatt 2014; Nunes et al. 2022). We sampled one leaf each from the top and bottom canopy of each of six trees per treatment during the leaf sampling campaign in the week beginning 25 July 2022. Epidermal impressions were obtained by applying clear nail varnish halfway on the abaxial surface of the leaves and allowed to dry for a few minutes. The dried nail varnish was gently peeled off using a pair of tweezers, ensuring that the peels were not stretched to distort the epidermal impressions. The epidermal peels were mounted on glass slides and analysed under a light microscope (Nikon LV100 ND; Nikon Corporation, Japan). Stomatal density, defined as the number of stomata per unit area (mm^2^) of the abaxial epidermis, stomatal (guard cell) length, pore length, and guard cell width were measured following protocols outlined in Franks and Beerling (2009) and Franks et al. (2014). Stomatal morphological traits were measured as the mean of 20 stomatal complexes (guard cell pairs) for each leaf at a 40× magnification. The anatomical maximum stomatal conductance to water vapour (*g*_wmax_, mol m^−2^ leaf s^−1^) was calculated using the equation by Franks and Beerling (2009):

(2) $$g_{\text{wmax}}=\frac{\frac{d_{w}}{v}\cdot D\cdot a_{\max}}{l+\frac{\pi }{2}\sqrt{a_{\max}/\pi }}$$

where *D* is stomatal density, *a*_max_ is the area of fully opened stomata, l is the depth of the stomata in meters and can be derived from the stomatal pore length. The terms $d_{w}$ and v are the diffusivity of water (m^2^ s^−1^) and the molar volume of air (m^3^ mol^−1^), respectively.

### 2.5 Micrometeorological Measurement at BIFoR FACE

Micrometeorological data, including canopy‐top air temperature (*T*_air_), relative humidity (RH), VPD, photosynthetic active radiation (PAR), precipitation, upper (ca. 10–40 cm) soil volumetric water content (VWC), and wind speed (WS) were utilized in this study (MacKenzie et al. 2021). Microclimatic data were collected at 25 m on the four met towers surrounding the experimental area (Figure S1), with the average value calculated for each time point. *T*_air_ was measured using Campbell Scientific 107 temperature thermistor probes (Logan, Utah, USA). PAR (μmol m^−2^ s^−1^) was measured using an LI190 by LI‐COR Biosciences (Lincoln, Nebraska, USA). The mean wind speed, WS (m s^−1^), was calculated as the average of 1‐min intervals using a WMT701 ultrasonic anemometer (Vaisala, Helsinki, Finland). Precipitation (mm) was measured using four TR‐525M rain gauges by Texas Electronics (Dallas, Texas). Microclimatic data were recorded at different resolutions but were organized into 10‐min time stamps to synchronize with the temporal resolution of the TIR camera. *T*_air_ and RH were used in calculating VPD (kPa) following Campbell and Norman (1998). Time series of the microclimate during the study period are shown in Figure S8.

### 2.6 Statistical Analyses

All analyses and figures were done using R 4.4.1 (R Core Development Team 2024). *T*_can_ for aCO_2_ and eCO_2_ oaks were analysed using 10‐min timestamp data. Data gaps due to equipment and software malfunctions were excluded.

Differences in *T*_can_ between aCO_2_ and eCO_2_ oaks were assessed using the daily maximum canopy temperature values. Due to the skewness of *T*_can_ data distribution (Figure S9D–F), Wilcoxon and Kruskal–Wallis rank‐sum tests were performed to evaluate differences between treatments (aCO_2_ and eCO_2_) and across years. Statistical significance was determined based on the Kruskal–Wallis test statistic (*χ* ^2^) and associated *p*‐value. Post hoc pairwise comparisons were conducted using Dunn's test with Bonferroni correction to identify differences across years.

LMA and LA were analysed with a linear mixed‐effects model using the ‘lmer’ package from the ‘lme4’ package in R. Fixed effects included CO_2_ treatment, canopy position, and their interaction, and month nested within year as a random effect. Tree‐level means (15 leaves per month) were used; assumptions were checked using visual inspection of residuals (normality and homoscedasticity), and significance was assessed at *α* = 0.05. Statistical comparisons of other leaf traits between treatments were performed using two‐sample *t*‐tests for single‐value comparisons, while the Wilcoxon rank‐sum test was used when normality assumptions were unmet.

Relationships between daytime canopy‐to‐air temperature (*T*_can_–*T*_air_) differences and microclimate variables, soil moisture as VWC (%), VPD (kPa), PAR (μmol m^−2^ s^−1^), and wind speed (WS, ms^−1^) were analysed using Generalised Additive Models (GAM) (mgcv package), which allow for non‐linear relationships (Wood 2017). High collinearity among predictors was identified using the concurvity() function in mgcv, and variables showing strong collinearity were excluded to optimise model performance.

## 3 Results

### 3.1 Impact of eCO_2_ on Canopy Temperature and Canopy‐to‐Air Temperature Difference

**FIGURE 2:**
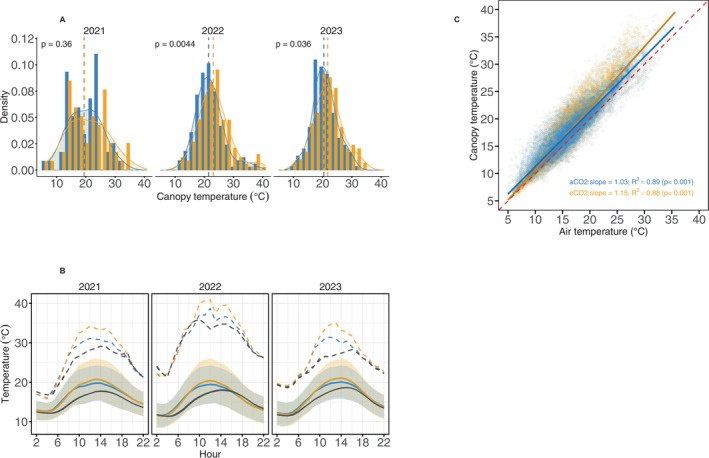
(A) Distribution of daytime daily maximum oak canopy temperatures (2021–2023) with density overlays; dashed vertical lines show treatment medians, and annotated *p*‐values indicate treatment differences. (B) Mean diurnal canopy and air temperatures for periods shown in Figure S9; solid lines show hourly means (shading = ±1 SD), and dashed lines show hourly maxima. (C) *T*_can_ versus *T*_air_ for eCO_2_ (orange) and aCO_2_ (blue); low‐opacity points represent individual observations, solid lines are regressions with 95% CIs, and the dashed red line indicates the 1:1 line.

Across all years, *T*_can_ was consistently higher under eCO_2_, with a median daily maximum of 22.5°C (IQR 19.2°C–25.7°C) under eCO_2_ compared to 21.2°C (IQR 18.6°C–23.9°C) under aCO_2_, a median difference of 1.3°C. Daily maximum *T*_can_ ranged from 10.9°C to 40.9°C under eCO_2_ and 11.6°C to 38.7°C under aCO_2_. Daily maximum canopy temperatures (Figure 2A) averaged 22.3°C ± 5.0°C in 2021, 22.5°C ± 5.2°C in 2022, and 21.4°C ± 4.5°C in 2023. A Kruskal–Wallis test confirmed significant interannual variation when aCO_2_ and eCO_2_ data were pooled (*χ* ^2^ = 18.6, df = 2, *p* < 0.001), with post hoc pairwise comparisons indicating that 2022 was significantly warmer than 2021 and 2023 (Table S5; Figure S5), reflecting the higher air temperatures that year. There was a significant effect of eCO_2_ on *T*_can_ in 2022 and 2023 (Figure 2A). In 2021, the *T*_can_ difference was not statistically significant, likely due to limited data availability early in the study period. However, analysis of high‐resolution 10‐min data for daylight hours (08:00–16:00) revealed significant treatment effects for all years (Figure S9), supporting a consistent warming of canopy temperatures under eCO_2_.

A linear regression of *T*_can_ versus *T*_air_ (Figure 2C) indicated that *T*_can_ in both aCO_2_ and eCO_2_ oaks was usually higher than *T*_air_ during the daytime. However, under eCO_2_, *T*_can_ increased more sharply with *T*_air_ (slope = 1.15; 95% CI: 1.14–1.15) than under aCO_2_ (slope = 1.03; 95% CI: 1.02–1.03), implying a more rapid warming of canopy leaves of eCO_2_ oaks with rising temperature.

Canopy temperature patterns of oaks for all years followed a strong diurnal pattern (Figure 2B). *T*_can_ temperature was not different between treatments during the nighttime and at certain times of the day. However, daytime *T*_can_ for eCO_2_ and aCO_2_ oaks peaked in the early afternoon (12:00–14:00), with eCO_2_ trees consistently having higher maximum *T*_can_ values than aCO_2_ trees. Post hoc comparisons showed that eCO_2_ canopies were warmer than both aCO_2_ canopies and *T*_air_ (*p* < 0.001 for each), and aCO_2_ canopies were warmer than *T*_air_ (*p* < 0.001).

**FIGURE 3:**
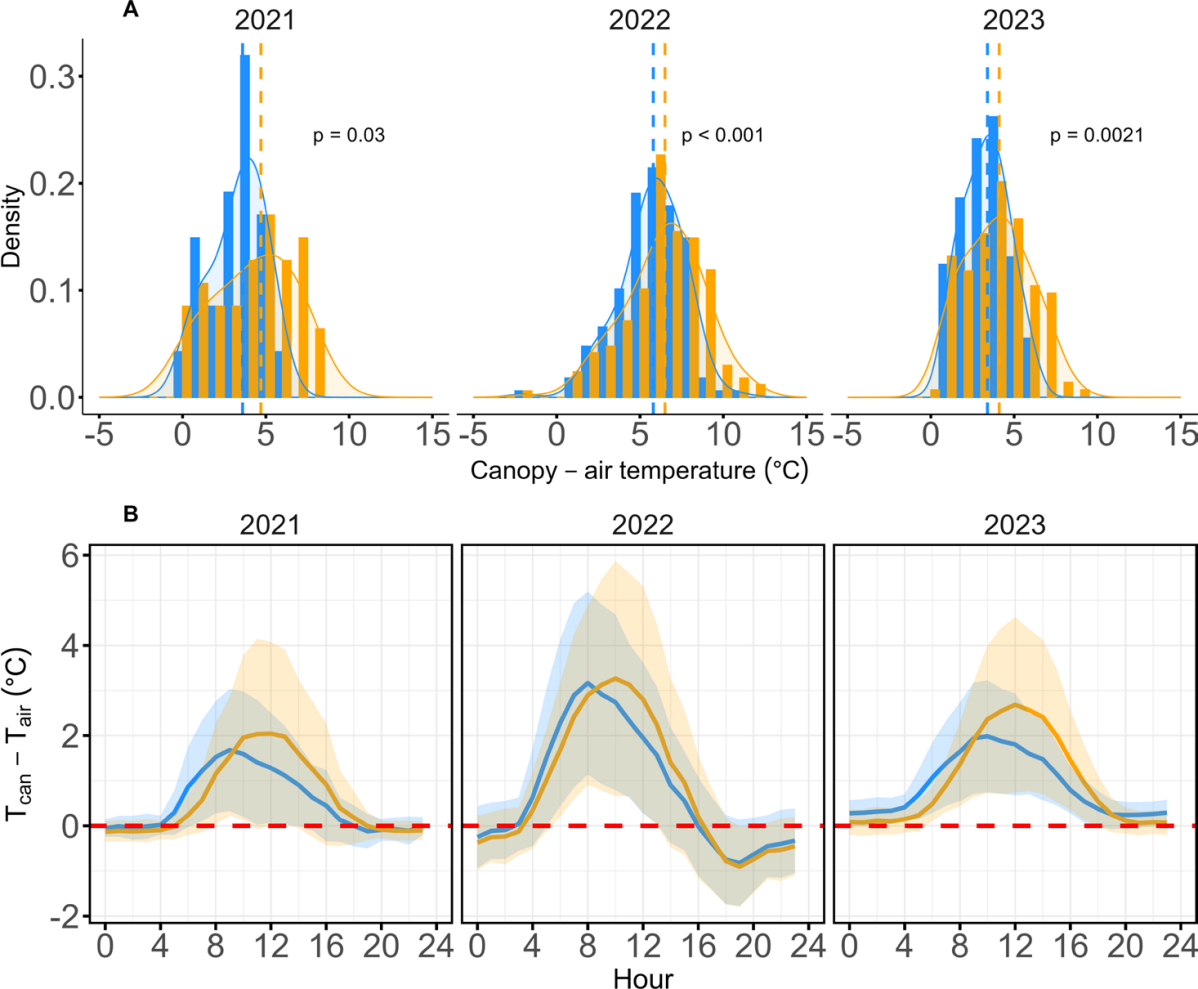
(A) Distribution of daily maximum *T*_can_−*T*_air_ differences (2021–2023), with density overlays and dashed lines indicating the median values per treatment. (B) Diurnal cycles for *T*_can_−*T*_air_ using hourly maximum values across all years; solid coloured lines show treatment means, shaded areas represent ±1 SD, and the dashed red line marks *T*_can_ = *T*_air_.

Across treatments, mean daily maximum *T*_can_–*T*_air_ differences were higher under eCO_2_ (6.8°C ± 2.2°C) than aCO_2_ under aCO_2_ (5.9°C ± 1.8°C), with differences statistically significant in all years (Figure 3A). The distributions of daily maximum *T*_can_–*T*_air_ were skewed (Figure 3A) under both treatments, with higher positive differences observed for oaks under eCO_2_ compared with aCO_2_ oaks.

Diurnal *T*_can_–*T*_air_ pattern (Figure 3B) revealed rapid warming and cooling of oaks under aCO_2_, with peak *T*_can_–*T*_air_ deviations earlier in the day compared with oaks in eCO_2_. The pattern also showed that canopy temperatures substantially exceeded air temperatures, especially during midday and early afternoon, across the monitoring years, with *T*_can_–*T*_air_ excursions occasionally exceeding 10°C in 2022, the hottest year studied and, indeed, the fifth hottest summer in the UK climate record from 1884 up to and including 2025 (Press Office 2025).

### 3.2 Impact of eCO_2_ on Leaf Thermal Traits

**FIGURE 4:**
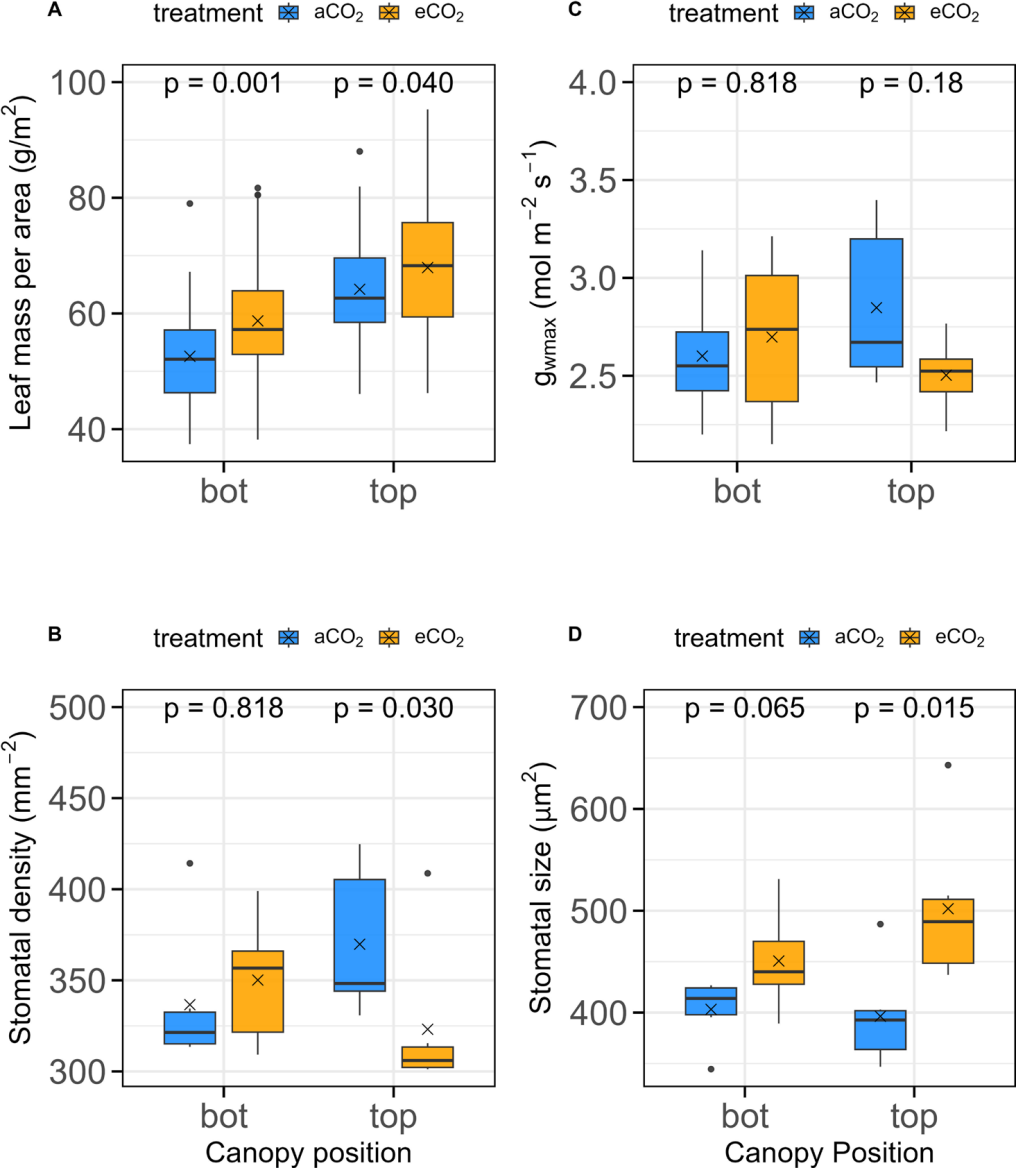
Boxplots of leaf trait differences in oaks under aCO_2_ and eCO_2_ conditions for bottom (bot) and top leaves. (A) Leaf mass per area (LMA) for top and bottom canopy leaves. (B) Stomatal density for top and bottom canopy leaves. (C) Anatomical maximum stomatal conductance to water vapour (*g*_wmax_). (D) Stomatal size for top and bottom canopy leaves (calculated as the product of stomatal length and width). Stomatal anatomical measurements were made on 24 leaf samples collected in July 2022.

Leaf mass area (LMA) was significantly higher under eCO_2_ compared to aCO_2_ (*p* < 0.001; Figure 4A; Table S6), with an estimated increase of 6.12 g/m^2^ (9.6% increase). Canopy position also had a significant effect (*p* < 0.001; Table S6), with top canopy leaves showing an average LMA increase of 11.58 g/m^2^ compared to bottom canopy leaves. There was no significant interaction between treatment and canopy position (*p* = 0.35), indicating that the effect of CO_2_ on oak LMA was consistent across canopy levels. There was no significant effect of eCO_2_ on LA (*p* = 0.51), nor a significant interaction between treatment and canopy (*p* = 0.28; Figure S5; Table S7). However, LA was significantly affected by canopy position (*p* = 0.034), with top canopy leaves being on average smaller than those from the bottom canopy.

Additionally, in the sample studied (*n* = 24 leaves), stomatal density in eCO_2_ decreased significantly by 12.6% (*p* = 0.03; Figure 4B) in top canopy leaves. Stomatal length (Figure S5) and size increased under eCO_2_, with a significant 15.7% enlargement observed in the top canopy leaves (*p* = 0.02; Figure 4D) and an overall 11.2% increase across both top and bottom canopy leaves. Despite the significant decrease in stomatal density in top canopy leaves in eCO_2_, this did not translate into a significant reduction in anatomical stomatal conductance (*g*_wmax_) of leaves between treatments (*p* = 0.18; Figure 4C).

### 3.3 Impact of eCO_2_ on Relationships Between Canopy‐to‐Air Temperature Differences and Microclimatic Variables

**FIGURE 5:**
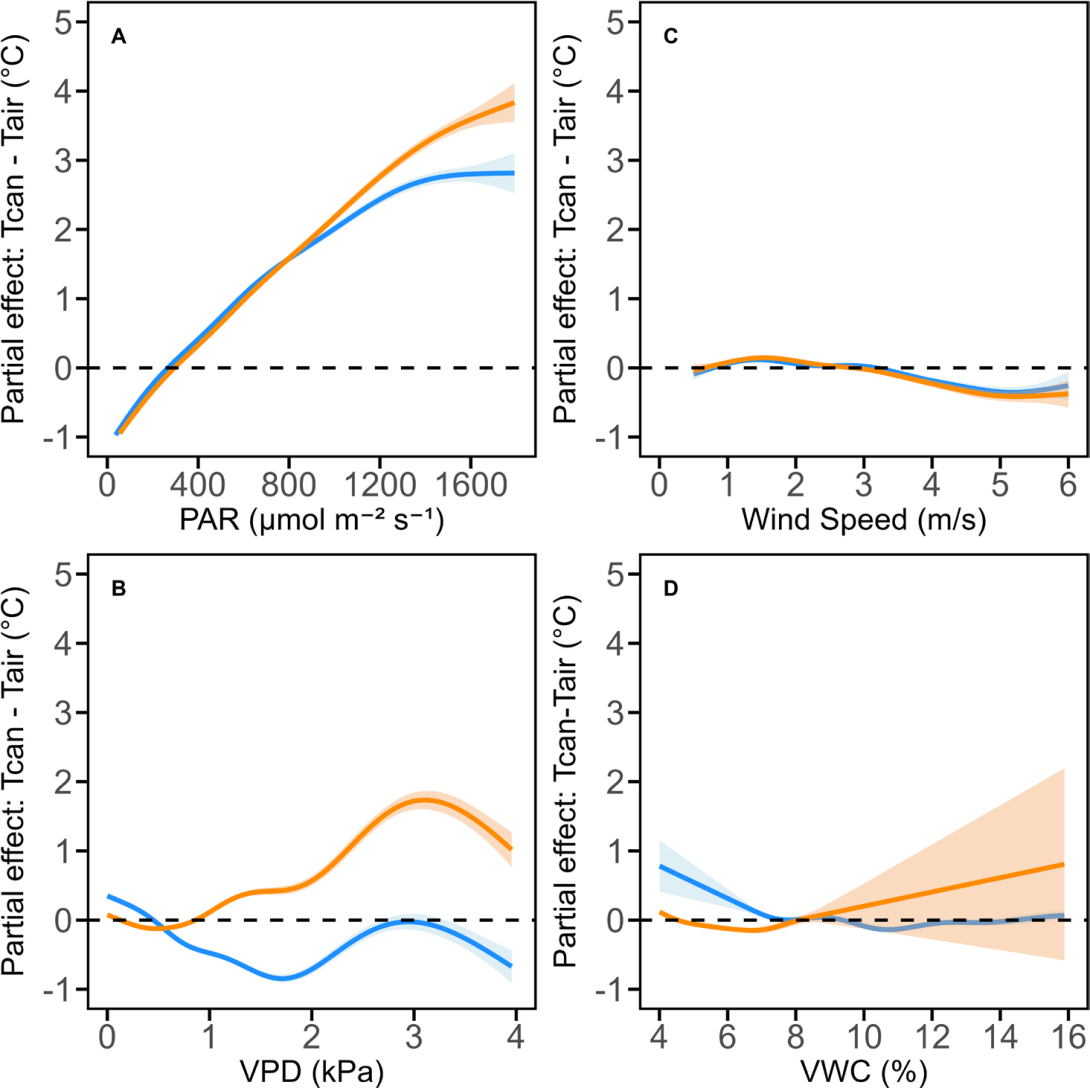
Partial effect plots from the GAM analysis showing the relationship between *T*_can_−*T*_air_ and microclimatic variables of mature oaks under eCO_2_ (orange) and aCO_2_ (blue). Shaded areas indicate 95% confidence intervals. Variables include: (A) PAR (μmol m^−2^ s^−1^), (B) VPD (kPa), (C) WS (m/s), and (D) VWC (%) for soil depth 10–40 cm. The small fraction (< 1%) of data beyond WS of 6 m/s and PAR of 1800 μmol m^−2^ s^−1^ was excluded to highlight relevant patterns within the observed ranges.

The GAM smoothing functions in Figure 5A–D for oaks in eCO_2_ and aCO_2_ conditions demonstrated that *T*_can_–*T*_air_ responded significantly to all the microclimatic variables examined in this study. Among these variables, VPD and PAR were the primary drivers of variation in *T*_can_–*T*_air_, exhibiting larger partial effects for both eCO_2_ and aCO_2_ oaks compared to WS and VWC. PAR was consistently positively correlated with *T*_can_–*T*_air_ under aCO_2_ and eCO_2_ (Figure 5A). Under eCO_2_, the strongest partial effect was observed, with *T*_can_–*T*_air_ exceeding 3°C above PAR of 900 μmol m^−2^ s^−1^, indicating consistently higher *T*_can_–*T*_air_ across PAR compared to aCO_2_.

The relationship between *T*_can_–*T*_air_ and VPD was more complex (Figure 5B). Under aCO_2_, *T*_can_–*T*_air_ was negatively correlated with VPD up to about 1.5 kPa, indicating that increasing VPD stimulated transpiration and enhanced cooling until higher VPD thresholds were reached (Figure 5B). In contrast, under eCO_2_, *T*_can_–*T*_air_ effectively remained relatively constant when VPD was below 0.5 kPa and only began to increase at higher VPD levels. At VPD ≥ 1 kPa, the aCO_2_ canopy maintained a lower *T*_can_–*T*_air_ than the eCO_2_ canopy, consistent with stronger cooling. These results suggest that the aCO_2_ canopy displays greater transpiration and a more dynamic stomatal conductance response to changing VPD. Wind speed (WS) and VWC exhibited weak negative relationships with *T*_can_–*T*_air_ under both aCO_2_ and eCO_2_ conditions (Figure 5C,D).

## 4 Discussion

### 4.1 Effect of eCO_2_ on Canopy Temperature and Canopy‐to‐Air Temperature Difference of Mature *Q. robur*

Our results show that eCO_2_ significantly increases canopy temperatures in mature oaks (Figure 2A). This effect seems primarily driven by altered stomatal conductance, which limits transpirational cooling, a mechanism well documented in the literature (Ainsworth and Rogers 2007; Bernacchi et al. 2007; Leuzinger and Körner 2007) and previously reported at our current study site (Gardner et al. 2021, 2023). Lower stomatal opening under eCO_2_, although weak, may contribute to improved water‐use efficiency through reduced transpiration. However, decreased transpiration causes a larger portion of incoming solar radiation to be radiated as sensible heat in the top canopy leaves. While studies on *T*_can_ in mature forests are scarce, with most research being conducted on crops, our empirical findings on *T*_can_ under similar elevated CO_2_ levels are consistent with the limited literature (Bernacchi et al. 2007; Hussain et al. 2013; Leuzinger and Körner 2007). Indeed, among the cited studies, only the study by Leuzinger and Körner (2007) assesses mature forest conditions.

Elevated CO_2_ treatments consistently resulted in higher daytime canopy temperatures than ambient conditions, with the strongest contrasts observed during years with extreme heat events (Figure 2A,B). These results highlight that interannual variability in environmental conditions, particularly atmospheric warming, can amplify the thermal impact of eCO_2_ on plant or forest canopies (Bernacchi et al. 2007; Warren, Norby, and Wullschleger 2011). In 2022, for example, possible limited transpirational cooling of oaks under eCO_2_ coupled with exceptionally high ambient temperatures led to more frequent and intense canopy warming excursions beyond 30°C (Figure S9). This suggests that as background temperatures rise under climate change, the warming effect of eCO_2_ on canopy temperatures may intensify (Figures 2A and 3A). Gardner et al. (2021) similarly observed reduced carbon assimilation during dry years at the same site, supporting our results that interannual environmental variability, such as drought, plays a critical role in shaping tree physiological and thermal responses.

Relationship of *T*_can_ versus *T*_air_ (Figure 2C) also showed slopes greater than one in both treatments, indicating that canopy temperature increases more steeply than air temperature by 3% under aCO_2_ and 14% under eCO_2_. This pattern suggests that oak canopies, especially under eCO_2_, experience less daytime cooling consistent with leaf‐level data (Figures S6 and S7). This is particularly evident within the 20°C–30°C air temperature range, which is optimal for photosynthesis (Kumarathunge et al. 2019). Our results reinforce evidence that daytime canopy temperatures already exceed air temperatures and are likely to continue to do so under a warming climate (Guo et al. 2023; Still et al. 2022). Conversely, these observations are at odds with the leaf homeothermy hypothesis advanced by Mahan and Upchurch (1988) and Michaletz et al. (2015).

Across the years, we observed a consistent shift in the diel pattern of peak in *T*_can_–*T*_air_ for oaks with peak values occurring later in the day under eCO_2_, as shown in Figure 3B. The reasons behind this are not clear, but it could again be a consequence of reduced stomatal conductance under eCO_2_, diurnal changes in boundary layer conductance, and leaf structural differences. Other reasons could be a change to the timing of sap flow through the tree, as the aCO_2_ trees have greater cooling than eCO_2_ under high VPD (Figure 5B), and so perhaps they are maintaining sap flow through the afternoon when eCO_2_ trees are not. In both aCO_2_ and eCO_2_ conditions, canopies warmed markedly during the early morning hours, a pattern driven by intense radiative heating, minimal transpirational cooling, and consistently low wind speeds, causing low boundary layer conductance (Still et al. 2022). eCO_2_ canopies, which possess a higher leaf mass per area (LMA) and, hence, greater thermal inertia, warmed more gradually in the morning under low wind conditions and maintained increased temperatures into the evening. While high LMA typically buffers leaves from extreme temperatures (Leigh et al. 2012), this protective effect seems to be outweighed by the heating caused by reduced transpiration under eCO_2_ conditions. Together, these effects delay the time at which sensible heat dominates, shifting the maximum *T*_can_–*T*_air_ later for eCO_2_ relative to aCO_2_.

Small, and occasionally negative, nighttime *T*_can_–*T*_air_, as observed especially in 2022 (Figure 3B), is unsurprising (due to the absence of solar radiation), for reasons well known in the production of ground frosts and dew (e.g., Monteith and Unsworth 2013). Similar patterns have been reported in both ponderosa pine forests (Kim et al. 2016) and tropical forests (Pau et al. 2018), resulting from the strong radiative heat loss from canopies to space (Jones 2014; Doughty and Goulden 2008) with implications for turbulence and canopy‐air exchange (Bannister et al. 2022). This nocturnal cooling, further enhanced by low humidity and stable boundary layers, results in condensation on leaf surfaces (Monteith and Unsworth 2013; Still et al. 2021), alleviating stress following extreme daytime heat.

### 4.2 Effects of Leaf Trait Alterations From eCO_2_ and Its Influence on Canopy Temperature

Stomatal anatomy plays a crucial role in regulating gas exchange between leaves and the atmosphere, influencing transpiration and carbon assimilation (Lawson and Vialet‐Chabrand 2019; Woodward and Kelly 1995; Woodward et al. 2002). In this study, there was a tandem adjustment in stomatal traits with increases in stomatal size and a reduction in stomatal density in top canopy leaves (Figure 4B,D) without a significant difference in *g*_wmax_. Such compensatory anatomical mechanisms maintain stable *g*_wmax_ (Nunes et al. 2022), and likely optimise gas exchange while enhancing water‐use efficiency under eCO_2_ conditions (De Boer et al. 2011; Franks and Beerling 2009; Hetherington and Woodward 2003; Lammertsma et al. 2011). Although fewer stomata might be expected to limit anatomical stomatal conductance in top canopy leaves (*g*_wmax_, Figure 4C) and thus latent heat loss, the presence of larger stomata, individually allowing greater gas exchange per stomate, may not fully compensate for the overall reduction in conductance, which is determined by both stomatal size and density (Dow et al. 2014; Lawson and Blatt 2014). This may be because, within the same species, larger stomata are often associated with slower response times compared to smaller stomata (Kardiman and Ræbild 2018) due to their lower surface‐to‐volume ratio, which could limit their capacity to adjust to environmental fluctuations faster (Lawson and Blatt 2014; McAusland et al. 2016; Nunes et al. 2022). Slow stomata thus could have contributed to the lag in leaf warming and sustained higher temperatures in oak leaves under eCO_2_ (Figure 2B) and thus the shift observed for their diurnal *T*_can_–*T*_air_ (Figure 3B). Together, these results support the interpretation that anatomical changes in stomata under elevated CO_2_ are not always functionally limiting, possibly due to compensatory scaling of other traits or post‐developmental physiological regulation (Saban et al. 2019).

Studies have reported increased LMA under eCO_2_, leading to thicker canopy leaves (Kovenock and Swann 2018), an observation reported in the early years of the BIFoR FACE experiment for top canopy leaves of oaks (Gardner et al. 2022). This increase in LMA could modify the radiative properties of canopy leaves by increasing their thermal time constant while also enhancing radiation absorption (Kovenock and Swann 2018), causing leaves to warm more slowly in the morning and retain heat longer during the day (Figures 1 and 3B). Indeed, Vogel (2009) and Leigh et al. (2012) also reported that higher LMA extends the period over which leaves absorb and store heat, especially when leaf cooling by transpiration is limited. Increased LMA could thus increase the thermal and radiative properties of the canopy by increasing the thermal time constant, meaning that leaves warm up more slowly in the morning and retain heat longer during the day, as noted above.

Other important leaf thermal traits not examined in our study, but known to influence canopy temperature, include leaf angle, trichomes, venation, emissivity, absorptance, transmittance, leaf cuticle, and reflectivity (Richardson et al. 2021; Vinod et al. 2023). Possible alterations in these unexamined traits under different CO_2_ growth conditions may interact with biophysical factors to affect canopy temperature, warranting their investigation in future studies.

### 4.3 Influence of Microclimate on Canopy‐to‐Air Temperature Difference

Our generalised additive model (GAM) analysis results show that the *T*_can_–*T*_air_ in mature *Q. robur* is influenced by VPD and PAR. While PAR contributes to leaf heating, VPD governs the potential for evaporative cooling, and their effects on *T*_can_–*T*_air_ vary under aCO_2_ versus eCO_2_ conditions.

We observed a positive relationship between PAR and *T*_can_–*T*_air_ for eCO_2_ and aCO_2_ (Figure 5A). High PAR increases the radiation load on canopy leaves, elevating *T*_can_ and, consequently, *T*_can_–*T*_air_ (Guo et al. 2023; Fauset et al. 2018; Jones 2014), particularly under eCO_2_, where leaf absorptance may be enhanced as shown schematically in Figure 1. Under aCO_2_, high PAR may promote unrestrained stomatal opening (with available soil moisture), driving transpiration and leaf cooling through latent heat loss. However, under eCO_2_ conditions, the typical stomatal response to high light may be dampened due to reduced stomatal conductance. As a result, even when PAR is high, eCO_2_ canopies may not increase transpiration as much to counterbalance the increased radiation load, resulting in a higher *T*_can_–*T*_air_ difference (Figure 5A). Our results align with empirical evidence showing that reduced stomatal conductance under eCO_2_ limits transpirational cooling under high PAR, leading to greater canopy warming relative to aCO_2_ conditions (Zhu et al. 2017).

VPD is a critical driver of transpiration. When soil moisture is sufficient, and plant hydraulics are intact, an increase in VPD typically stimulates higher transpiration rates, which cools the leaves through latent heat loss (Drake et al. 2013); hence the generally negative partial contribution of VPD to *T*_can_–*T*_air_ under ambient conditions (aCO_2_; Figure 5B). However, if soil water becomes limiting and VPD exceeds a certain threshold, stomatal conductance declines to conserve water (Grossiord et al. 2020), reducing transpiration and leading to higher *T*_can_–*T*_air_ differences. Under eCO_2_, the effect is magnified because it compounds the reduction in stomatal conductance, even at baseline VPD (Ainsworth and Rogers 2007; Novick et al. 2024). Consequently, for a given high VPD, the eCO_2_ oak canopies experience a larger *T*_can_–*T*_air_ difference than the oaks under aCO_2_ (Figure 5B), as sufficient transpiration to counteract the increased evaporative demand is limited. These results align with a study by Novick et al. (2024), who reported that rising VPD not only dehydrates leaves but also compounds the effects of reduced transpiration under eCO_2_. Theoretical evidence from Kirschbaum and McMillan (2018) also corroborates our results, emphasizing that rising CO_2_ levels reduce stomatal conductance, which has a transpiration‐suppressing effect that may outweigh the increased evapotranspiration driven by higher temperatures from future climate change (Zhu et al. 2017).

Wind plays a role in determining leaf boundary layer thickness by influencing convective heat loss (Monteith and Unsworth 2013; Vinod et al. 2023). The large, closed, and complex canopy structure of mature oaks creates thick boundary layers around leaves, contributing to the low *T*_can_–*T*_air_ sensitivity to wind speed under both treatments (Figure 5C). Under eCO_2_, where latent cooling is reduced, low wind speeds may exacerbate the decoupling of canopy temperature from air temperature, although this could be compensated by increasing LMA, as reported by some studies (Leigh et al. 2012; Vogel 2009).

Canopy‐to‐air temperature differences were minimally sensitive to soil moisture changes (Figure 5D), likely because soil was generally well‐watered (likely due to high precipitation throughout monitoring years) and the anisohydric stomatal behaviour of oaks. Anisohydric species maintain relatively stable stomatal conductance even as soil moisture fluctuates (Yi et al. 2020), sustaining a consistent latent heat exchange and limiting significant variations in *T*_can_–*T*_air_. Additionally, deep‐root water access in mature oaks, as reported in Yi et al. (2020), likely buffered *T*_can_–*T*_air_ against changes in soil water availability, reducing sensitivity in both treatments. However, under eCO_2_ conditions, anisohydric behaviour could be compromised during extreme heat events, despite potential water savings from enhanced water‐use efficiency from the former (Kirschbaum and McMillan 2018; Novick et al. 2024).

## 5 Conclusion

This 3‐year thermal infrared imaging study demonstrates that eCO_2_ (ca. 150 ppm above ambient) increases daily maximum canopy temperatures in mature *Q. robur* by approximately 1.3°C. Our data suggest that this rise in canopy temperature may be driven by reduced stomatal conductance and increased radiative absorptance, potentially due to higher LMA, which together affect the canopy's energy balance. Such changes may increase the risk of photosynthetic impairment and other physiological processes under future warming scenarios.

The observed distinct diurnal temperature patterns further underscore the influence of leaf trait acclimations and environmental factors on canopy thermal dynamics. Alterations in leaf structural and physiological characteristics, such as leaf mass per area, stomatal anatomy, and stomatal conductance, collectively contribute to higher canopy temperatures. Reduced stomatal conductance under eCO_2_ could likely decrease transpiration, reducing the humidity of the lower troposphere, which could subsequently affect precipitation patterns in downwind regions. These findings highlight how future CO_2_ conditions may intensify thermal stress in temperate forests, influencing water and carbon cycles and potentially impacting forest resilience.

## Author Contributions

**William Hagan Brown:** conceptualization, data curation, formal analysis, investigation, methodology, project administration, software, visualization, writing – original draft, writing – review and editing. **Emanuel Gloor:** formal analysis, methodology, software, supervision, validation, visualization, writing – review and editing. **Ralph Fyfe:** formal analysis, methodology, project administration, software, supervision, visualization, writing – review and editing. **A. Rob MacKenzie:** funding acquisition, methodology, project administration, supervision, visualization, writing – review and editing. **Nicholas J. Harper:** data curation, resources, software. **Peter Ganderton:** data curation, resources, software. **Kris Hart:** project administration, resources. **Giulio Curioni:** data curation, resources, software. **Susan Quick:** data curation, investigation, writing – review and editing. **Scott J. Davidson:** supervision, visualization, writing – review and editing. **Emily Yetton:** formal analysis, investigation. **Jen L. Diehl:** formal analysis, software. **Sophie Fauset:** conceptualization, formal analysis, funding acquisition, investigation, methodology, project administration, resources, supervision, validation, visualization, writing – review and editing.

## Conflicts of Interest

The authors declare no conflicts of interest.

## Supporting information


**Data S1:** gcb70565‐sup‐0001‐DataS1.pdf.

## Data Availability

The raw canopy temperature dataset from the BIFoR FACE facility has been deposited at the NERC Environmental Information Data Centre (EIDC). The DOI for the data is: https://doi.org/10.5285/71e0cc1b‐59bd‐4b7d‐994c‐fa4b1c9689d9. Additionally processed canopy temperature, trait and microclimate data are openly available in figshare at https://doi.org/10.6084/m9.figshare.30233401.
