# Supplementary material for: Elevated CO2 Increases the Canopy Temperature of Mature Quercus robur (Pedunculate Oak)
Source: Glob Chang Biol. 2025 Nov 5;31(11):e70565. doi: 10.1111/gcb.70565 (PMC12587106; doi:10.1111/gcb.70565)
Supplement: Supplementary file 1 — Data S1: gcb70565‐sup‐0001‐DataS1.pdf. [file GCB-31-e70565-s001.pdf]

Supplementary Information

Elevated CO_2 increases the canopy temperature of mature *Quercus robur* (pedunculate oak)

William Hagan Brown[1], Ralph Fyfe[1], Emanuel Gloor[3], A. Rob MacKenzie[2], Nicholas J. Harper[2], Peter Ganderton[1], Kris Hart[2], Giulio Curioni[2], Susan Quick[2], Scott J. Davidson[1], Emily Yetton[1], Jen L. Diehl[4,5], Sophie Fauset[1].

Tables

Table S1. Annual microclimate summary

Table S2. Annual canopy temperature summary

Table S3. GAM Results: T_{can} – T_{air} by CO_2 treatment

Table S4. Linear Models: T_{can}, T_{leaf} versus T_{air}

Table S5. Dunn post-hoc test showing canopy temperature results.

Table S6. Fixed effects summary from mixed model showing results of eCO_2 on LMA.

Table S7. Fixed effects summary from mixed model showing results of eCO_2 on leaf area

Figures

Figure S1. Study site map and camera view

Figure S2. Camera field of view in LabVIEW

Figure S3. Thermal infrared camera correction workflow

Figure S4. Comparison of raw and corrected thermal camera temperature measurements against black-body thermocouple reference temperatures

Figure S5. Boxplots of leaf area differences in oaks under aCO_2 and eCO_2 conditions for bottom (bot) and top canopy leaves.

Figure S6. Histogram comparisons of the impact of eCO_2 on the T_{leaf} versus T_{air} relationship as measured using thermistors and TIR camera

Figure S7. Linear regression comparisons of the impact of eCO_2 on the T_{leaf} versus T_{air} relationship as measured using

Figure S8. Daily micro-meteorological data at the BIFoR FACE site during canopy temperature monitoring periods.

Figure S9. Time series of mean oak canopy temperatures for monitoring years.

Table S1. *Annual summary statistics of daily microclimate data for years where canopy temperature (T_{can}) data were available (blue shaded regions in Figure 1). Variables include air temperature (T_{air}), precipitation (pcpn), relative humidity (rh), photosynthetically active radiation (PAR), wind speed (WS), and vapour pressure deficit (VPD). Reported values represent annual means, standard deviations, maxima, minima, or totals as appropriate.*

year	T_{air} mean	T_{air} _sd	T_{air} _max	Tair_min	pcpn_total	rh_mean	rh_min	PAR_mean	PAR_max	VPD_mean	VPD_sd	VPD_max
2021	15.7	2.9	29.1	4.9	91.2	82.2	65.8	291.2	2098.5	0.37	0.21	2.38
2022	14.9	3.4	35.9	4.4	500.7	77.1	37.6	311.3	2211.5	0.46	0.36	4.54
2023	15.3	2.7	27.8	4.3	278.0	78.9	55.4	340.5	2124.3	0.41	0.21	2.26

To support accurate image analysis and enhance the utility of the thermal infrared (TIR) data, I suggest pairing the TIR camera with an RGB camera configured to capture the same field of view (FOV). This dual-camera setup will be highly beneficial during post-processing, as the high-resolution RGB imagery can assist with more precise delineation of regions of interest (ROIs) within the thermal imagery. Moreover, this approach will enable a better contextual interpretation of thermal data and improve the reliability of pixel-level analyses, particularly in complex canopy environments or heterogeneous landscapes, such as the tropics.

Table S2. *Statistical summary of annual diurnal hourly maximum canopy temperature (T_{can}) under ambient and elevated CO_2 treatments. Values include the median, mean, standard deviation (sd), first quartile (Q1), third quartile (Q3), and interquartile range (IQR) for each treatment and year.*

**year**	**treatment**	**median_Tcan**	**mean_Tcan**	**sd_Tcan**	**Q1**	**Q3**	**IQR**
**2021**	aCO2	24.95	24.2	5.7	17.7	29.8	12.1
**2021**	eCO2	25.7	25.2	6.9	17.2	32.4	15.2
**2022**	aCO2	31.0	30.1	5.7	25.1	35.6	10.5
**2022**	eCO2	32.2	31.3	6.7	25.2	37.5	12.4
**2023**	aCO2	24.4	25.1	4.3	21.4	29.2	7.8
**2023**	eCO2	24.7	26.1	5.4	21.7	31.6	9.9

Table S3 (A) & (B). *Summary of Generalized Additive Model (GAM) results assessing the effects of microclimatic variables on maximum canopy-to-air temperature difference (T_{can} – T_{air}) under* ***(A)*** *ambient CO_2 (aCO_2) and* ***(B)*** *elevated CO_2 (eCO_2) conditions. The model includes smooth terms for wind speed (WS_met), vapour pressure deficit based on air temperature (VPD_tair), photosynthetically active radiation (PAR_met), and soil water content at a depth of 10–40 cm (swc_mean_10to40cm).*

(A)

```
summary(gam_model_eCO2)


Family: gaussian

Link function: identity


Formula:

Tcan_Tair_max_v2 ~ s(WS_met) + s(VPD_Tair) + s(PAR_met) +
s(swc_mean_10to40cm)

Parametric coefficients:

            Estimate Std. Error t value Pr(>|t|)

(Intercept) 0.958341   0.009266   103.4   <2e-16 ***

---

Signif. codes:  0 ‘***’ 0.001 ‘**’ 0.01 ‘*’ 0.05 ‘.’ 0.1 ‘ ’ 1

Approximate significance of smooth terms:

                      edf Ref.df       F p-value

s(WS_met)           4.809  5.888   40.40  <2e-16 ***

s(VPD_Tair)         5.862  7.030   60.31  <2e-16 ***

s(PAR_met)          7.097  8.143 2025.88  <2e-16 ***

s(swc_mean_10to40cm) 5.601  6.720   31.82  <2e-16 ***

---

Signif. codes:  0 ‘***’ 0.001 ‘**’ 0.01 ‘*’ 0.05 ‘.’ 0.1 ‘ ’ 1

R-sq.(adj) = 0.684   Deviance explained = 68.5%

-REML = 17312  Scale est. = 1.0331    n = 12032

>
```

(B)

```
> summary(gam_model_aCO2)


Family: gaussian
Link function: identity


Formula:
Tcan_Tair_max_v2 ~ s(WS_met) + s(VPD_Tair) + s(PAR_met) + 
s(swc_mean_10to40cm)
Parametric coefficients:
            Estimate Std. Error t value Pr(>|t|)    
(Intercept)  0.88841    0.00877   101.3   <2e-16 ***
---
Signif. codes:  0 ‘***’ 0.001 ‘**’ 0.01 ‘*’ 0.05 ‘.’ 0.1 ‘ ’ 1


Approximate significance of smooth terms:
                       edf Ref.df       F p-value    
s(WS_met)            5.799  6.944   25.40  <2e-16 ***
s(VPD_Tair)          6.748  7.856  132.90  <2e-16 ***
s(PAR_met)           7.412  8.377 1853.74  <2e-16 ***
s(swc_mean_10to40cm)  5.937  7.106   23.42  <2e-16 ***
---
Signif. codes:  0 ‘***’ 0.001 ‘**’ 0.01 ‘*’ 0.05 ‘.’ 0.1 ‘ ’ 1


R-sq.(adj) =  0.607   Deviance explained = 60.8%
-REML =  16656  Scale est. = 0.92537   n = 12032
```

Table S4 (A). Summary of linear regression results examining the relationship between canopy temperature (T_{can}) and air temperature (T_{air}) under aCO_2 (A) and eCO_2 (B) conditions .

Treatment	Intercept	Slope	95% CI (Slope)	Std. Error	R^2	DF
eCO_2	−0.794	1.149	[1.144, 1.155]	0.00276	0.8757	24,532
aCO_2	0.842	1.028	[1.023, 1.032]	0.00224	0.8948	24,651

Table S4 **(B)** Linear regression models (**Figure S7 A**) of leaf temperature (T_{leaf}) against air temperature (T_{air}) under eCO_2 (plot 5) and aCO_2 (plot 6), measured using thermistors.

Treatment	Slope	Std. Error	t value	p value	R^2	DF
eCO_2	1.10285	0.00324	340.4	< 0.001	0.9879	1424
aCO_2	1.04793	0.00276	380.0	< 0.001	0.9902	1424

Table S4 **(B)** Linear regression models (**Figure S7 B**) of canopy temperature (T_{can}, measured with TIR camera) as a function of air temperature (T_{air} from met towers) under eCO_2 and aCO_2 conditions.

Treatment	Slope	Std. Error	t value	p value	R^2	DF
eCO_2	1.09112	0.00277	394.0	< 0.001	0.9912	1377
aCO_2	1.06536	0.00245	435.8	< 0.001	0.9928	1374

Table S5. Dunn post-hoc pairwise comparisons among years for daily maximum canopy temperature (max_Tcan) pooled across aCO_2 and eCO_2 treatments. The Z statistic, raw p – value (P.unadj), and Bonferroni – adjusted p – value (P.adj) are reported for each year pair. After Bonferroni correction, 2022 was significantly warmer than both 2021 and 2023 (P.adj < 0.05), whereas 2021 and 2023 did not differ.

**Comparison**	**Z**	**P.unadj**	**P.adj**
2021 - 2022	-3.926211	$8.629434e^{-05}$	0.000258883
2021 - 2023	-1.631734	$1.027355e^{-01}$	0.308206636
2022 - 2023	3.041917	$2.350768e^{-03}$	0.007052305

Table S6. Summary of fixed effects from the linear mixed-effects model testing the influence of CO_2 treatment, canopy position, and their interaction on leaf mass per area (LMA). The model included a random intercept for month nested within year: LMA ~ treatment * canopy + (1 | year/month). Estimates are presented as mean ± standard error. Significant effects ($p < 0.05$) are indicated in bold.

**Fixed effect**	**Estimate ± SE**	**df**	**t-value**	**p-value**
(Intercept)	52.61 ± 2.10	4.12	25.09	**< 0.001 *****
Treatment (eCO_2)	+6.12 ± 1.80	221.02	3.40	**0.00081 *****
Canopy (top)	+11.58 ± 1.87	221.02	6.20	**< 0.001 *****
Treatment × Canopy	−2.39 ± 2.55	221.04	−0.94	0.350

Table S7. Summary of fixed effects from the linear mixed-effects model testing the influence of CO_2 treatment, canopy position, and their interaction on leaf area. The model included a random intercept for month nested within year: leaf area ~ treatment * canopy + (1 | year/month). Estimates are presented as mean ± standard error. Significant effects ($p < 0.05$) are indicated in bold.

**Fixed effect**	**Estimate ± SE**	**df**	**t-value**	**p-value**
(Intercept)	373.11 ± 26.37	3.03	14.15	**0.00073 *****
Treatment (eCO_2)	+11.76 ± 17.73	221.01	0.66	0.508
Canopy (top)	−39.28 ± 18.40	221.01	−2.14	**0.0339 ***
Treatment × Canopy	+27.30 ± 25.13	221.02	1.09	0.279

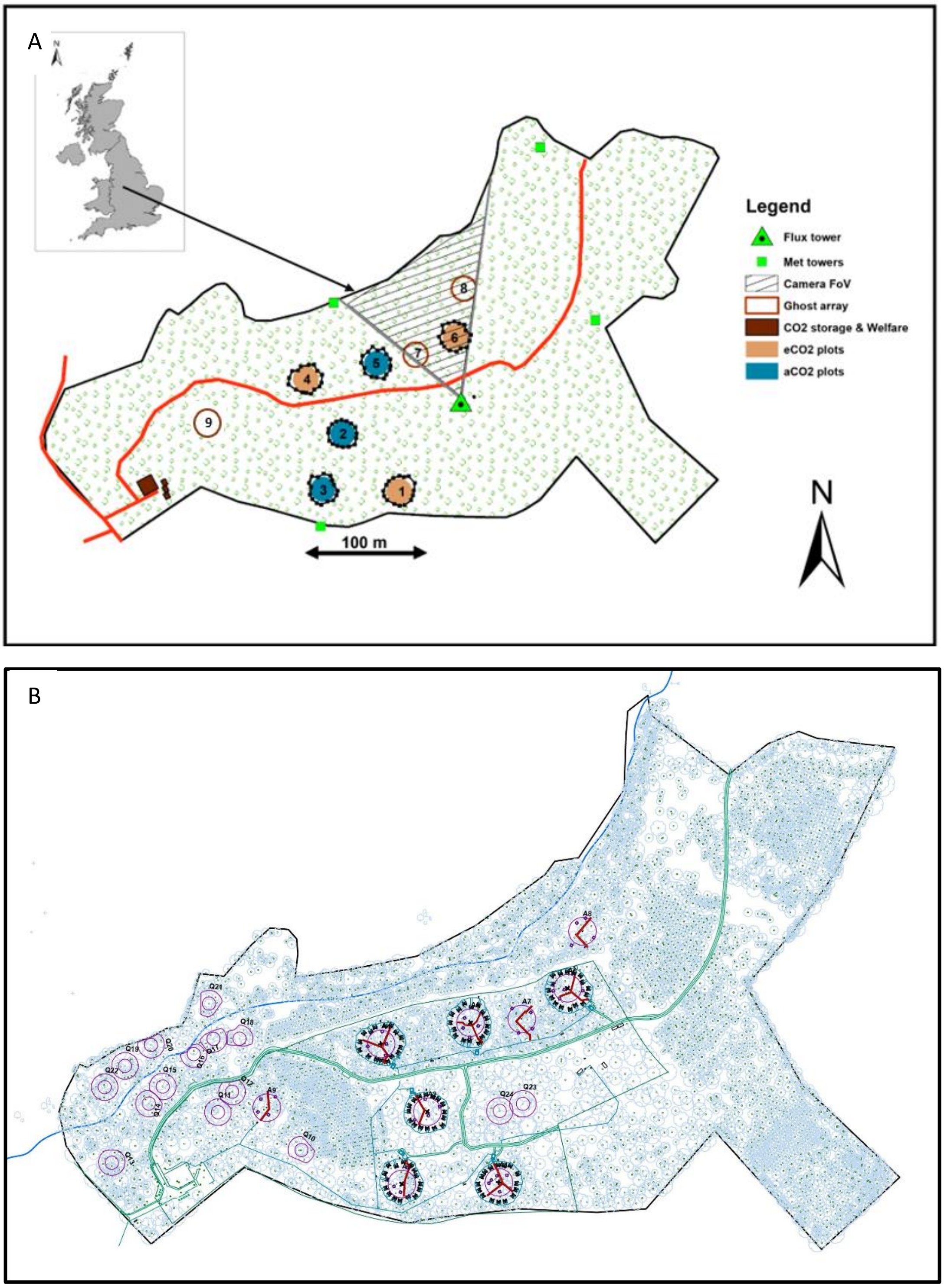

Figure S1: (A) Map of study site show treatment, control and infrastructure plots. The shaded areas indicate the camera's field of view (FOV). (B) Detailed map showing tree densities across the study site (Credit: Birmingham Instute of Forest Research, University of Birmingham).

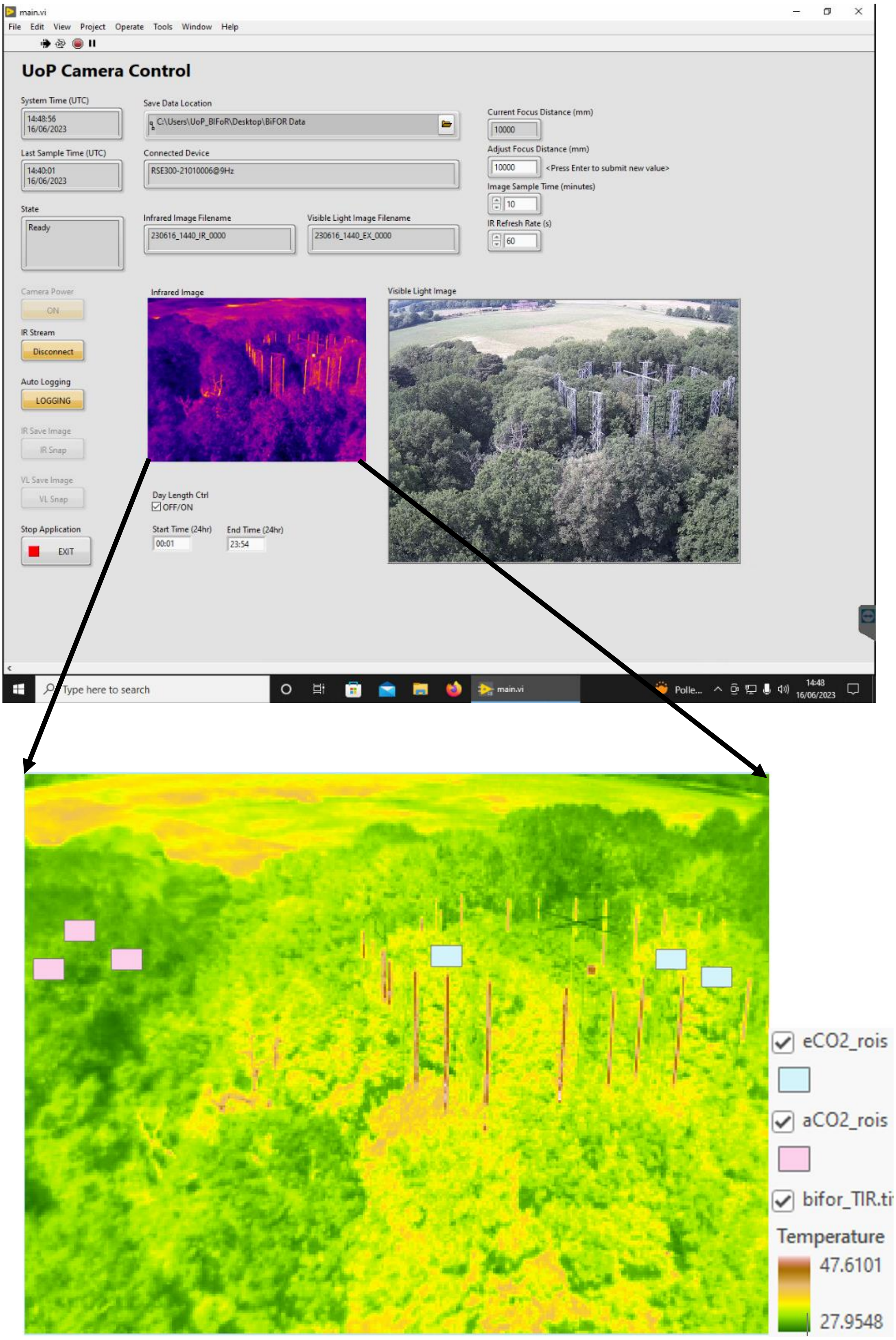

Figure S2: Field of View for TIR camera and visible light camera on camera control software, LABVIEW. ROIs shown are only indicative: actual analysis used four ROIs each per treatment.

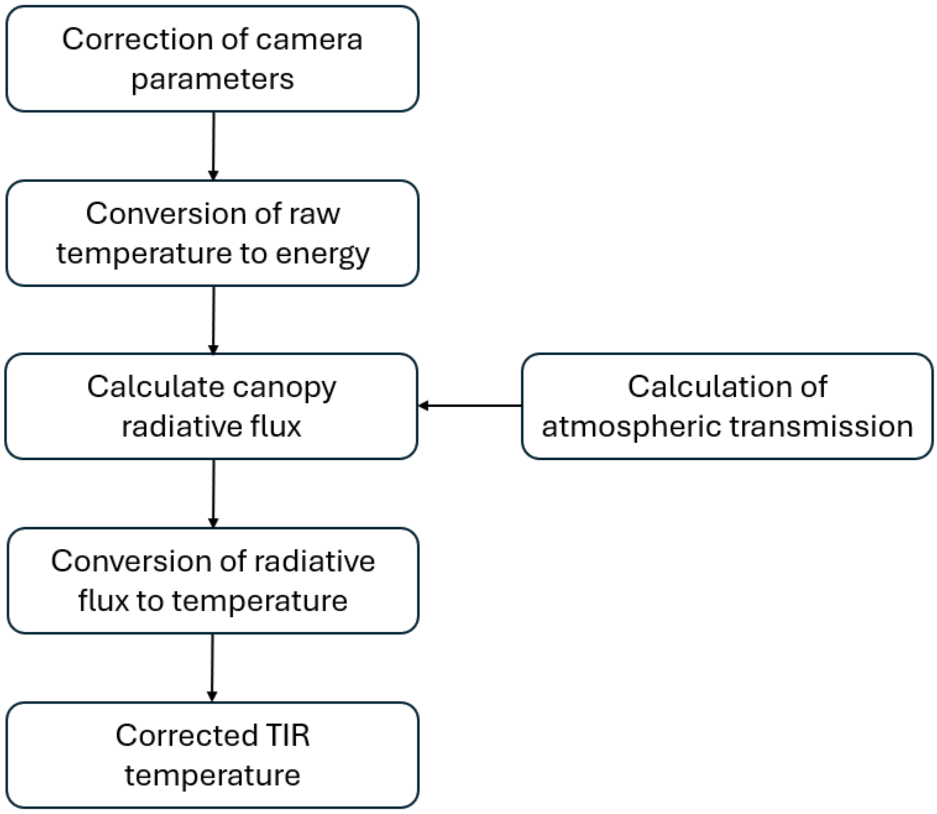

Figure S3: Workflow for correcting raw thermal camera data using site-based microclimatic factors

A

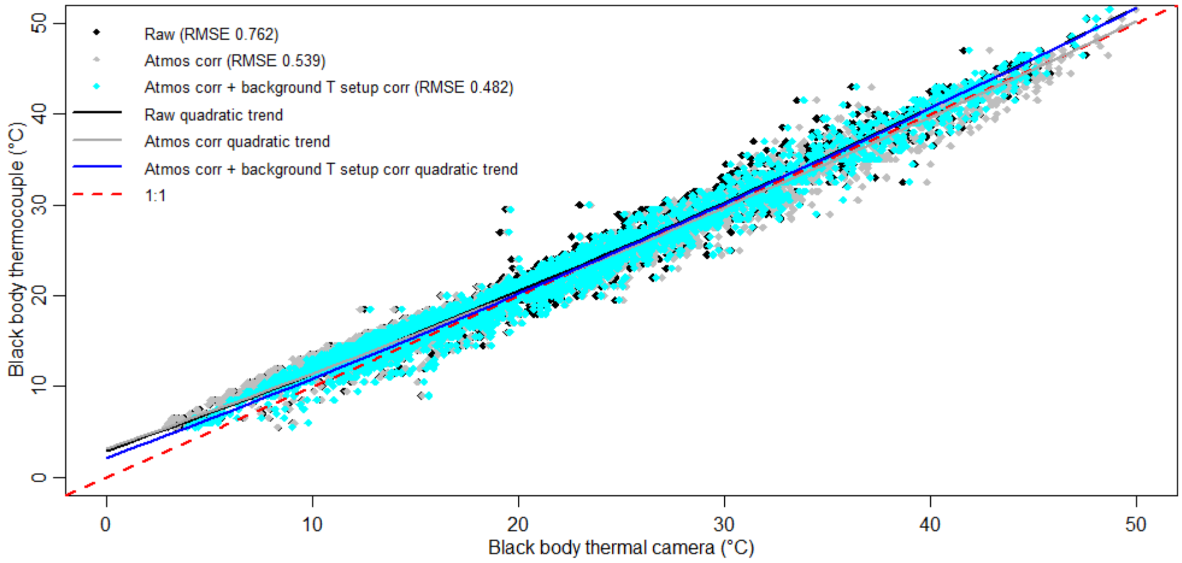

B

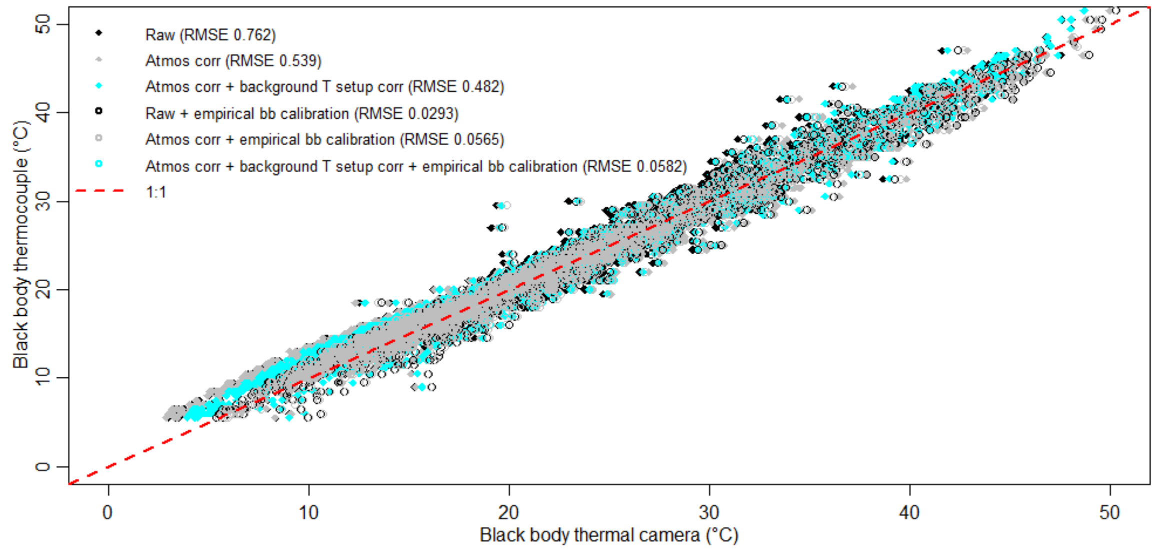

Figure S4. Comparison of raw and corrected thermal camera temperature measurements against black-body thermocouple reference temperatures. (A) Quadratic relationships between measurements and the reference show that atmospheric corrections (cyan) perform best at higher temperatures, whereas combined atmospheric and background setup corrections (blue) improve performance at lower temperatures. Raw measurements (black) generally exhibit the poorest fit, with the highest RMSE values. (B) The addition of an empirical black-body calibration step markedly reduces RMSE, bringing corrected measurements (cyan and grey circles) into close alignment with the reference across the full temperature range (note the cyan and grey circles are very similar, therefore overlaid). The dashed red line indicates the 1:1 reference line.

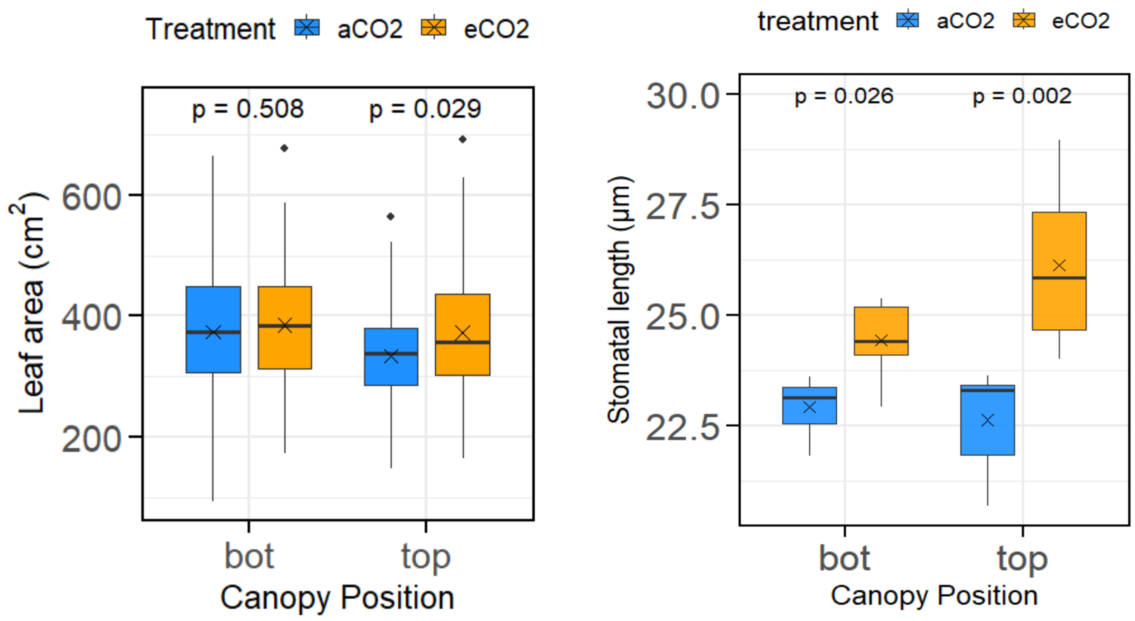

Figure S5. (A) Boxplots of leaf area differences in oaks under aCO_2 and eCO_2 conditions for bottom (bot) and top canopy leaves. Leaf area represents monthly means (June to August) for 2021 to 2023. (B) Stomatal length for bottom and top canopy leaves was measured in July 2022. Figure

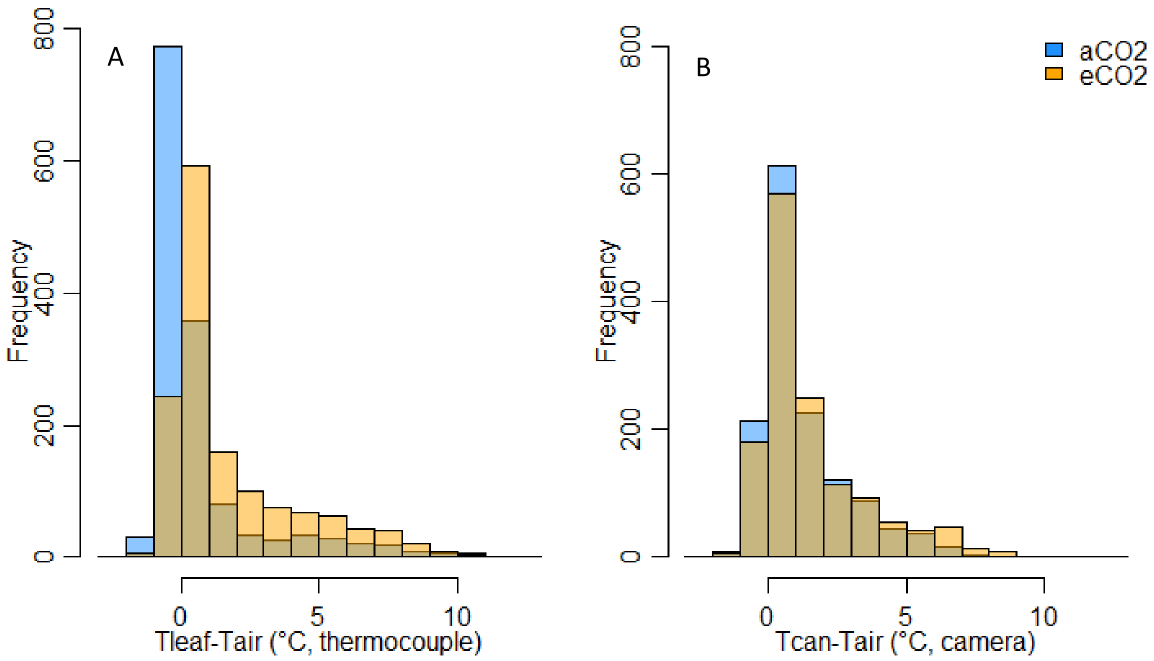

Figure S6. Comparison of CO_2 effect on **(A)** Tleaf-Tair measured with thermocouples on leaves in aCO_2 Array 5 and eCO2 Array 6, with **(B)** Tcan-Tair measured with a thermal camera viewing ghost Array 7 and eCO_2 Array 6.

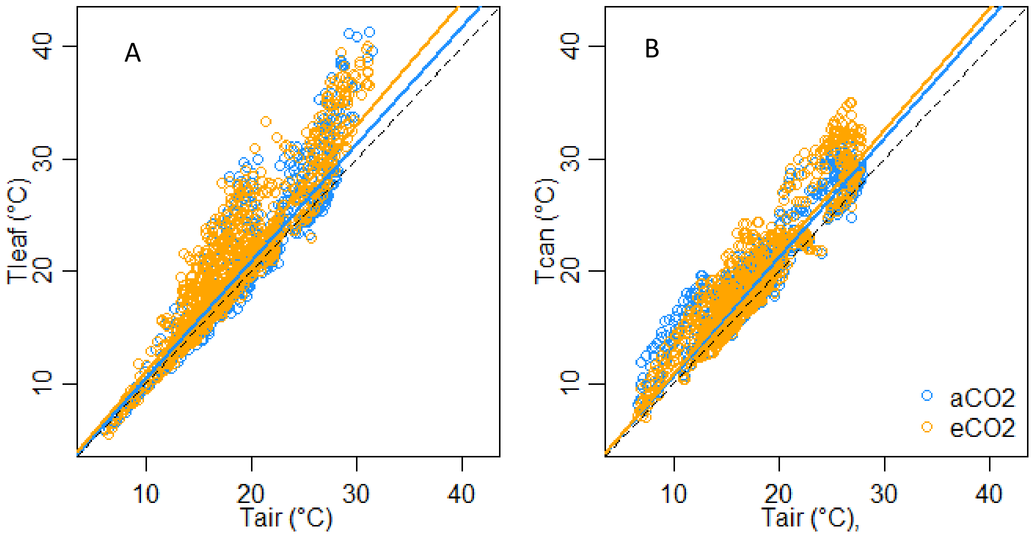

Figure S7. Comparison of the impact of eCO_2 on the T_{leaf} versus T_{air} relationship as measured using **(A)** thermocouples in aCO_2 Array 5 and eCO_2 Array 6, and **(B)** Tcan versus Tair as measured using a thermal camera viewing ghost Array 7 and eCO_2 Array 6. The regression equations are **(A):** eCO_2: $T_{leaf} = 1.103*T_{air}$, aCO_2: $T_{leaf} = 1.048*T_{air}$; **(B)** eCO_2 :$T_{can} = 1.091*T_{air}$, aCO_2 :$T_{can} = 1.065*T_{air}$. The origin of the regression was forced through zero for a simpler comparison of the eCO_2 treatment effect.

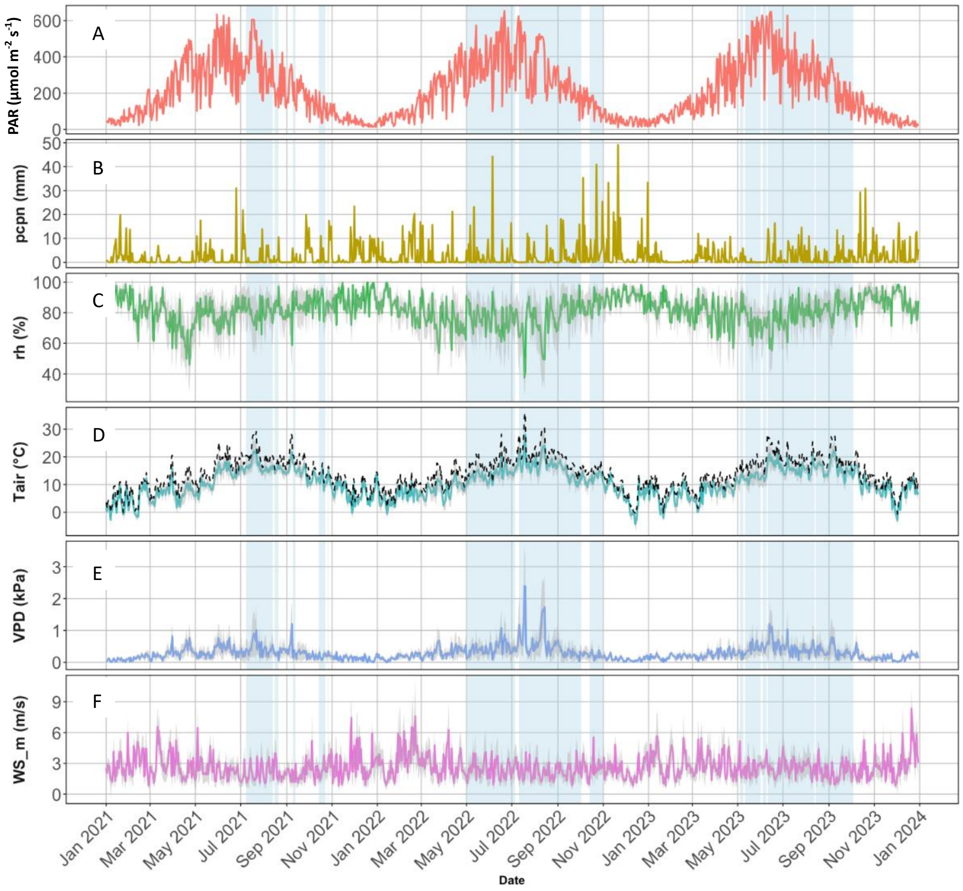

Figure S8. Daily micro-meteorological data at the BIFoR FACE site during canopy temperature monitoring periods. Blue-shaded areas indicate the TIR monitoring window for each year. Solid lines represent daily (A) mean PAR (µmol m^{-2} s^{-1}), (B) total precipitation (mm), (C) mean RH (%), (D) mean air temperature (°C), (E) mean VPD (kPa), and (F) mean wind speed (ms^{-1}). The black dashed line in panel (D) shows the daily maximum air temperature. The grey ribbon around the mean (panels C, D, E and F) represents the standard deviation.

0.00

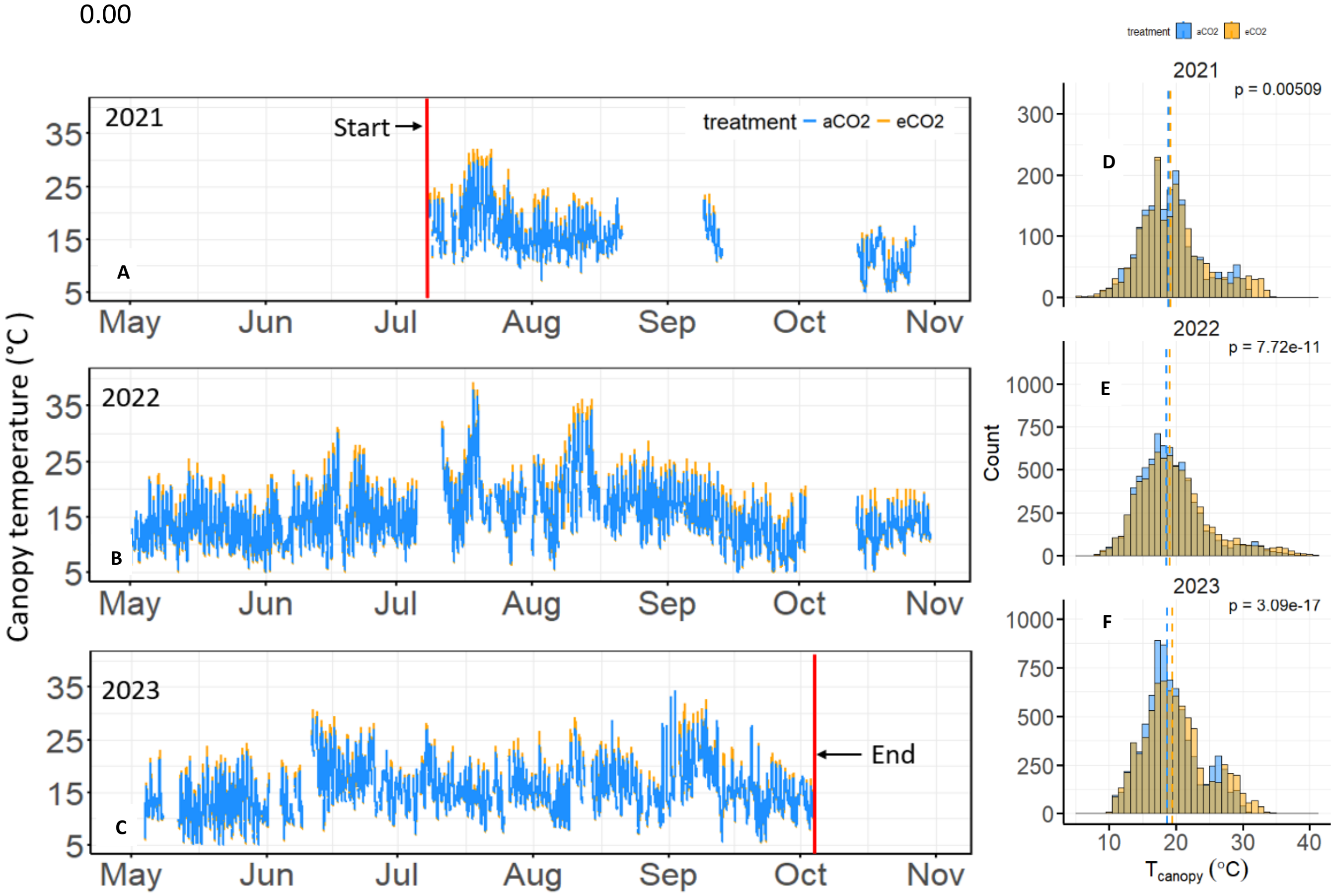

Figure S9. (A to C) Time series of mean oak canopy temperatures for monitoring years. Thick lines represent the 10-minute timestamps for temperatures for both treatments. The red vertical lines in (A) and (C) indicate the start and end of the monitoring period for this study. Plots 'D' to 'F' are histograms of daytime (09:00 to 16:00 hours) canopy temperature for aCO_2 and eCO_2 trees with dashed lines marking the median values per treatment. P-values shows the significance in the difference between treatments within each year based on Wilcoxon tests.
